# Pericytes: Intrinsic Transportation Engineers of the CNS Microcirculation

**DOI:** 10.3389/fphys.2021.719701

**Published:** 2021-08-23

**Authors:** Ahmed M. Eltanahy, Yara A. Koluib, Albert Gonzales

**Affiliations:** ^1^Department of Physiology and Cell Biology, School of Medicine, University of Nevada, Reno, NV, United States; ^2^Tanta University Hospitals, Faculty of Medicine, Tanta University, Tanta, Egypt

**Keywords:** pericytes, blood-brain barrier, Alzheimer’s disease, stroke, neurovascular coupling, functional hyperemia, regenerative medicine

## Abstract

Pericytes in the brain are candidate regulators of microcirculatory blood flow because they are strategically positioned along the microvasculature, contain contractile proteins, respond rapidly to neuronal activation, and synchronize microvascular dynamics and neurovascular coupling within the capillary network. Analyses of mice with defects in pericyte generation demonstrate that pericytes are necessary for the formation of the blood-brain barrier, development of the glymphatic system, immune homeostasis, and white matter function. The development, identity, specialization, and progeny of different subtypes of pericytes, however, remain unclear. Pericytes perform brain-wide ‘transportation engineering’ functions in the capillary network, instructing, integrating, and coordinating signals within the cellular communicome in the neurovascular unit to efficiently distribute oxygen and nutrients (‘goods and services’) throughout the microvasculature (‘transportation grid’). In this review, we identify emerging challenges in pericyte biology and shed light on potential pericyte-targeted therapeutic strategies.

## Introduction

Our definition and understanding of pericytes within the capillary network have evolved from their initial discovery as simple mural cells with a distinctive “bump-on-a-log” morphology to a kind of a cellular jack-of-all-trades. Seminal studies published along the way have further supported important roles for these cells in capillary physiology in both health and disease. In the current review, we discuss the multi-faceted capabilities of pericyte within the capillary circulation and their essential role in the formation, preservation, and control of blood vessels and blood flow. We will also draw an anthropomorphic comparison of the pericyte’s role within the capillary network to that of a transportation engineer in building, maintaining, and regulating the throughways of transportation.

Eberth and Rouget were the first to describe a population of cells within the capillary circulation approximately 150 years ago (Stricker, 1871), initially naming them for their perivascular position, and later became the first to distinguish these cells from migratory leukocytes (Rouget, 1873). Fifty years later, Zimmermann renamed these cells ‘pericytes’ based on the location of the cell (*-cyte*) around (*peri-*) capillary vessels (Zimmermann, 1923). Since their initial discovery, pericytes have been credited with a vast array of physiological functions in the capillary vasculature. The Betsholtz’s and Daneman’s laboratories demonstrated that pericytes are critical for the normal formation of blood vessels and maintenance of the blood-brain barrier (BBB; Armulik et al., 2010; Daneman et al., 2010)—a highly selective, semipermeable border that separates the circulation from brain tissue. Around the same time, Attwell and colleagues reconfirmed the contractile nature of pericytes and branded them vascular ‘first responders’ in matching capillary blood flow to neuronal activity (Peppiatt et al., 2006; Hall et al., 2014). Since these formative papers, the function and definition of capillary pericytes have continued to be refined. Shih and colleagues (Hartmann et al., 2021) further classified pericytes into separate cellular subtypes, introducing the terms ‘ensheathing,’ ‘mesh,’ and ‘thin-stranded’ to describe points along the morphological continuum of capillary pericytes. Nelson and colleagues further provided novel observations establishing a post-arteriole transitional zone containing junctional contractile pericytes, demonstrating that capillary pericytes in this region control the morphology of capillary junctions and blood flow between branches to ensure efficient function of the capillary network and optimal perfusion of the brain (Gonzales et al., 2020). These observations and others from prominent investigators in the field (Fernández-Klett et al., 2010; Hamilton et al., 2010; Grubb et al., 2020) have lifted pericytes from obscurity and into the limelight as cells important for the construction, maintenance, and function of the capillary network. These “transportation engineer” functions of pericytes require the ability to efficiently coordinate functions, integrate signals, and instruct and otherwise communicate with different cell types in the surrounding microenvironment. In this review, we embellish on this conceptual role of pericytes as vascular transportation engineers of the central nervous system (CNS) in health and disease.

## Transportation Engineers of the Microvasculature

### Pericytes Fine Tune Blood Flow Within Capillary Networks to Match Neuroenergetics

The functional linkage between neuronal activity and blood flow, a process termed neurovascular coupling that is responsible for the activity-dependent increase in local blood flow (functional hyperemia), was previously thought to be solely mediated by changes in the tone of the smooth muscle cells that form a continuous layer around the endothelial cell lining of arterioles. Work by Attwell and colleagues pushed pericytes—the mural cells of the capillary network—into the neurovascular coupling conversation as major regulators of capillary diameter and blood flow that directly respond to neuronal activity (Hall et al., 2014). These observations were not without controversy. The contractile nature of pericytes had been previously debated by some (Hill et al., 2015), and others simply questioned the cellular identity of contractile mural cells, considering them to be smooth muscle cells. Currently, capillary pericytes are thought to represent a phenotypic continuum, with subtypes that differ in morphology, protein expression, and function showing a gradual transition with distance from the feeding arteriole. Recently, our lab and those of others (Hartmann et al., 2021; Hørlyck et al., 2021) have made efforts to functionally identify molecular players and contractile machinery within different subtypes of pericytes.

**FIGURE 1:**
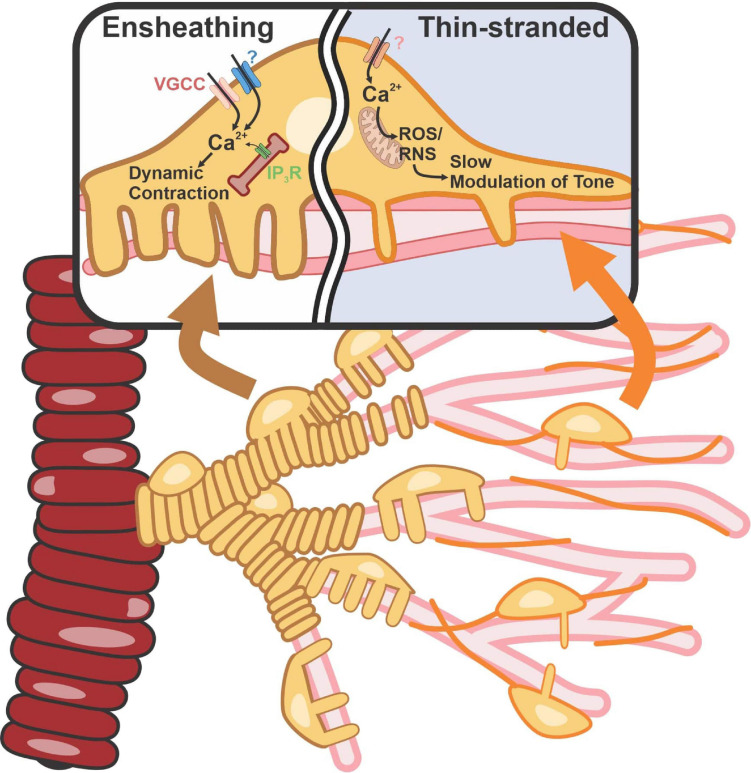
Contractile machinery in ensheathing vs capillary pericytes. Ensheathing pericyte’s morphology appears similar to that of SMCs but with a dense wrapping banding pattern of tightly packed projections from a single pericyte. They possess high amounts of α-SMA and are located on the transitional region. Capillary pericytes with a thin-strand morphology are located on high-order capillaries. These cells possess low amounts of α-SMA. Elevation of intracellular Ca^2+^ and free radical production activates Rho-kinase and polymerization and vasoconstriction. VGCC, voltage-gated calcium channel; ROS, reactive oxygen species; RNS, reactive nitrogen species.

The most widely accepted determinant of the different subtypes of pericytes is the expression of alpha-smooth muscle actin (α-SMA), the dynamically contractile, Ca^2+^-dependent actin isoform encoded by the *Acta2* gene. Pericytes also express moderate-to-high levels of genes encoding other contractile proteins, such as desmin and myosin light chain (Nehls and Drenckhahn, 1993; Sweeney et al., 2016). Efforts have also been made to identify the Ca^2+^ circuits in pericytes that contribute to the contraction of α-SMA–expressing pericytes within the post-arteriolar transitional region. Recent work from Gonzales et al. (2020) showed that pericytes can compartmentalize Ca^2+^ and contraction dynamics to the individual projections that wrap around different capillary branches. The compartmentalization of Ca^2+^-signaling domains to projections and the ability of these projections to contract independently of each other provides functional evidence that pericytes differ from vascular smooth muscle cells (VSMCs). Pericyte contraction also involves Ca^2+^ influx and intracellular release via L-type Ca^2+^ channels (LTCCs) and inositol triphosphate receptors (IP_3_Rs), respectively (Figure 1), not unlike VSMCs. But importantly, ryanodine receptors, a defining feature of all VSMCs, are functionally absent from pericytes and thus do not contribute to Ca^2+^ dynamics. Contractile junctional pericytes can also receive hyperpolarizing signals from downstream endothelial cells, leading to branch-specific dilatory responses that serve to increase the precision of blood flow delivery to active neurons (Cuevas et al., 1984; Gonzales et al., 2020; Kovacs-Oller et al., 2020). However, understanding the underlying biophysical and cellular mechanisms that enable pericyte projections to restrict Ca^2+^, electrical and contraction dynamics, and how consequent changes in the contractile state of daughter branches control junctional geometry to affect blood rheology and/or oxygen extraction, will require further investigation.

Numerous labs have reported that α-SMA expression in pericytes decreases in deeper reaches of the capillary tree, yet many other contractile proteins are still present in these cells (Lindskog et al., 2006; Billaud et al., 2017; Gonzales et al., 2020). A new study by Hartmann and colleagues using selective optical ablation or activation of capillary pericytes reported that capillary pericytes deeper in the capillary network exert both static and slow regulation of capillary diameter (Hartmann et al., 2021). In so doing, these cells can affect the resistance and flow through the vessel. This study also showed that this slow constriction of capillary vessels is inhibited by fasudil, a Rho kinase inhibitor used clinically as a vasodilator, suggesting the involvement of Rho kinase in this process (Figure 1). A role for Rho kinase in the maintenance and integrity of stress fibers through direct and/or indirect phosphorylation (Amano et al., 1996) and its possible regulation of non-muscle actin isoforms, β- and γ-actin (DeNofrio et al., 1989; Shum et al., 2011), could explain the slower kinetics of the response. Reactive oxygen species (ROS) link pericyte depolarization to Rho-kinase activation and, hence, vasoconstriction. ROS are critical in pericyte biology (Shah et al., 2013a). Specifically, it has been shown that pericyte contraction caused by ROS accumulation following brain ischemia/reperfusion injury leads to capillary constriction (Cai et al., 2017). With two broad functional subtypes—one capable of rapid, dynamic constriction and the other constricting through slow modulation of vessel tone—pericytes provide the means to control blood flow and the delivery of O_2_ and nutrients throughout the brain.

Quantitative neuroimaging techniques have revealed discrepancies between changes in cerebral blood flow and changes in the cerebral metabolic rate of oxygen (CMRO2), and thus the tissue oxygen extraction fraction (OEF; Angleys et al., 2018). Healthy brain tissue has excess oxygen delivery compared with utilization (Mintun et al., 2001). At rest, this excess oxygen diffuses back to the capillary, but, in neuronal activation, it may be used for oxidation without necessary increase of CBF. Taken together, if tissue demand increases or CBF decreases, tissue oxygen levels can be maintained in the viable range without the need for increased CBF. This ‘uncoupling’ of oxygen consumption from the degree of functional hyperemia reflects limitations in oxygen diffusion across microvascular walls (Tsai et al., 2003). OEF and blood flow are inversely related, highlighting a fundamental biophysical limitation in the ability of vasodilation to meet cellular oxygen demands (Jespersen and Østergaard, 2012; Østergaard, 2020). It has been hypothesized that such decreases in OEF could significantly reduce the metabolic benefits of a simple hyperemic response. Neural activity and hypoxia are not only followed by a general increase in blood flow, but also by a rapid redistribution of capillary flow to a more homogeneous flow pattern. Such homogenization of the capillary pattern is thought to increase OEF, raising the question of whether OEF can change independently of blood flow. This raises another interesting question: can contractile pericytes at capillary junctions determine tissue oxygenation through dynamic regulation of blood flow distribution—or redistribution—rather than by simply changing capillary resistance and overall blood flow?

The spatially restricted increase in cerebral blood flow that follows local neural activation constitutes the functional hyperemic response and forms the basis of functional magnetic resonance imaging (fMRI). The response occurs rapidly, with an onset time on a millisecond scale (Silva et al., 2000). Interestingly local neuronal stimulation and GABAergic and glutamatergic signaling were shown to cause retinal pericytes to dilate, implying a link between local inhibitory/excitatory balance and capillary hemodynamics and that there is an endogenously active neurotransmitter signaling to pericytes (Peppiatt et al., 2006). A generalized pericyte dilation would allow for more a homogeneous flow of red blood cells (RBCs) in response to local neurotransmitter release, allowing for higher OEF (Jespersen and Østergaard, 2012). The RBC velocity heterogeneity might be necessary for the redistribution of flow during activation. At baseline, it is known that capillary is very heterogeneous (Schmid et al., 2017). *In vivo* experiments showed that high and low flow capillaries respond differently to neuronal activity and that RBC velocity increases, as well as decreases, can be observed (Stefanovic et al., 2008). This redistribution of flow leads to a reduction of Capillary transit time heterogeneity (CTH). So, to upregulate the oxygen supply, at least two regulation mechanisms should be playing in synergism, namely CBF upregulation and capillary flow homogenization (Schmid et al., 2019). This can be more relevant at deeper cortical levels, where the feeding flow rate is lower and the CTH is larger leading to very effective extraction of oxygen is required, especially that RBCs saturation is lower in the deep cortical layers (Schmid et al., 2017, 2019). Accordingly, the topology of the microvascular network, for example, mass balance at the bifurcation, branch pressure gradient, and flow resistance can determine the ratio of RBCs distribution and heterogeneity of RBC capillary transit time (Schmid et al., 2019). Accordingly, constriction of functional thoroughfare routes with an ensuing redistribution of RBCs to capillaries with more homogenous transit times would result in a more efficient OEF (Østergaard, 2020). Therefore, pericytes are thought to regulate RBC distribution at capillary bifurcations and adjust local capillary diameter in response to local cellular needs (neuroenergetics), leading to subsequent changes in CTH and OEF that possibly contribute to the observed rapid variations in oxygen that serve to adapt to changing energy demands of the surrounding microenvironment.

### Pericytes Synchronize With Endothelial Cells to Support Effective Vascular Formation, Maintenance, and Control of Endothelial Specialization

Pericytes and endothelial cell dynamics are synchronized during capillary blood flow regulation (Armulik et al., 2005; Institoris and Gordon, 2021) and endothelial sprouting in angiogenesis (Coelho-Santos et al., 2021). Pericyte-endothelial physical interactions are hypothesized to be critical for providing microvascular wall stabilization by maintaining pericyte properties and/or holding them in “suspended animation” i.e., “the microvasculature can provide a niche that prevents differentiation of pericytes into mesenchymal cell derivative” (Bautch, 2011). Pericytes communicate with endothelial cells by direct contact in addition to multiple paracrine signaling pathways; for example, platelet-derived growth factor (PDGF), TGF-β, and Notch signaling (Bergers and Song, 2005). Gap junctions also provide direct communication between the cytoplasm of both cells facilitating the exchange of ions and molecules (Cuevas et al., 1984). Adhesion plaques anchor pericytes to endothelial cells, while peg-and-socket interdigitations penetrate deeply into the endothelial basement membrane supporting intercellular transmission of mechanical forces (Dessalles et al., 2021). Moreover, the contribution of the pericytes to the vascular basement membrane biochemical composition is considered significant due to the unique location of pericytes embedded in the endothelial basement membrane (Thomsen et al., 2017). Taken together, pericytes encounter “solid contact stresses” due to their direct physical interactions with the endothelial cell, being subjected to hemodynamic stresses and perivascular water flow/shear stresses (Dessalles et al., 2021). The transmural pressure difference “regulated by pericytes” leads to fluid crossing the vascular wall through endothelial cell-cell junctions and mechanically influencing pericytes and flowing around them. Pericyte-endothelial cell mechanical interaction was also shown to prevent endothelial cell-mediated extracellular matrix degradation, suggesting a possible mechanobiological mechanism for maintaining microvascular homeostasis.

Pericytes are not only involved in hemodynamic processes but also have an active role in the formation of the blood vessels on which they reside (Coelho-Santos et al., 2021). Pericytes proliferate during angiogenesis in the CNS and *in vitro*, and the lack of immature mesenchyme in the developing CNS implies that new pericytes develop mainly by the proliferation of pre-existing pericytes in this system. Pericytes can also modulate signaling processes activated by pro-angiogenic and pro-inflammatory cues to control angiogenesis in normal and pathological conditions (Kang et al., 2019). The expression of platelet-derived growth factor (PDGF)-B is not uniform in the developing endothelium, with tip cells expressing higher PDGF-B levels than stalk cells. Pericytes are immediately recruited to emerging angiogenic sprouts and lag only slightly behind the appearance of tip cells (Coelho-Santos and Shih, 2020; Coelho-Santos et al., 2021). Interestingly, NR2F2 (nuclear receptor subfamily 2, group F, member 2) also known as COUP-TFII (COUP transcription factor 2) (Qin et al., 2010), a major angiogenesis regulator during development and within the tumor microenvironment, has been shown to drive angiopoietin-1 (Ang-1) expression in pericytes; moreover, conditional ablation of *Nr2f2* in adult mice leads to compromised neo-angiogenesis in tumors and embryos (Suri et al., 1996; Pereira et al., 1999), supporting a role for pericytes in angiogenesis. Ang-1 expression in hematopoietic cells also appears to have a critical role in angiogenesis, highlighting a potential integrated role of hematopoietic cells and pericytes in this process (Takakura et al., 2000; Hattori et al., 2001). Expression of vascular endothelial growth factor receptor 1 (VEGFR1) in pericytes spatially restricts VEGF signaling, promoting endothelial sprouting during angiogenesis (Eilken et al., 2017). Blocking the interaction between proangiogenic growth factors and endothelial cells was shown to promote non-productive angiogenesis, manifesting as poor perfusion, hypoxia, and decreased tumor growth (Noguera-Troise et al., 2006), suggesting an interesting approach for treating tumors that are resistant to conventional therapies. Non-productive angiogenesis around Aß plaques was recently identified in Alzheimer’s disease (Alvarez-Vergara et al., 2021). Taken together, these observations suggest that normalization of the microvasculature is a viable therapeutic approach in clinical settings of cancer biology, stroke, and neurodegeneration, as will be discussed in detail below.

Endothelial cells are highly specialized across vascular beds under both physiologic and pathologic conditions (Cleuren et al., 2019). A gradual cellular, biochemical and functional transition along the vascular tree has been well-established. Do pericytes play a role in this microenvironmental heterogeneity? In capillaries, there is only one basement membrane between endothelial cells and astrocytic endfeet, termed the ‘fused gliovascular membrane’ (Bechmann et al., 2007). However, the absence of a fused gliovascular membrane at the post capillary venules might drive the BBB-typical specialization of the endothelium. Solute diffusion is regulated at the capillary level (Groothuis et al., 2007), whereas leukocyte recruitment occurs preferentially in post-capillary venules. As we will discuss later, pericytes can remodel the perivenular basement membrane representing a path of “least resistance” for immune cell trafficking (Wang et al., 2012; Thomsen et al., 2017).

### Pericytes Coordinate Immune Cells and Regulate the Perivascular Microenvironment

“Pericyte sensome” is a novel term that we define as the range of ligands sensed by pericytes. Pericytes are strategically positioned to sense inflammation and send activation/inhibition signals within the local microenvironment. The extravasation of White Blood Cells (WBCs) through blood vessels is a key step in host defense (Kolaczkowska and Kubes, 2013). Certain subtypes of pericytes were discovered to be highly responsive sensory cells that interpret inflammatory signals and send out specific instructions to infiltrating WBCs (Alon and Nourshargh, 2013). What are the mechanisms underlying the immunological learning in-motion phenomenon? (Alon and Nourshargh, 2013) and how do pericytes impose immune privilege on the CNS microvasculature?

The concept behind “CNS immune privilege” imposes strict regulations on immune cell trafficking between the periphery and parenchyma (Mastorakos and McGavern, 2019) which has been recently questioned after the discovery of the Dural lymphatic system (Louveau et al., 2015). For that reason, it is hypothesized that increased pericyte density within the brain likely contributes to the defense of the brain from patrolling leukocytes and pathogens, acting as a “biophysical shield” to maintain tissue homeostasis (Armulik et al., 2011). On the other hand, pericyte-induced remodeling of the perivenular basement membrane represent a path of least resistance for immune cell trafficking (Wang et al., 2012; Thomsen et al., 2017). In response to a stimulus, (for example, inflammation or infection), pericytes express cytokine receptors and toll-like receptors (TLRs) and release cytokines and chemokines (Rafalski et al., 2018). It has been shown that innate immune cells that interact with neuron-glial antigen 2-expressing (NG2^+^) pericytes can reach the site of necrosis earlier, supporting the proposition that pericytes ‘instruct’ leukocytes by improving their motility programs (Proebstl et al., 2012; Alon and Nourshargh, 2013). Pericytes are a major source of interleukin-6 (IL-6) in the brain (Fabry et al., 1993; Matsumoto et al., 2018), and IL-6 signaling has been shown to provide a molecular ‘switch’ that governs immune cell trafficking across the microvasculature (Chen et al., 2006; Stark et al., 2013). Notably, this process is facilitated by febrile temperatures, which are closely linked to the inflammatory response, as heat is one of the four cardinal signs of inflammation that confer a survival benefit during infection (Evans et al., 2015). Although febrile temperatures can improve the vascular delivery of inflammatory cells to tissues by regulating hemodynamic parameters such as vasodilation and blood flow, the direct role of fever/temperature in modulating pericytes is not clearly understood.

Taken together, pericytes govern the size and location of leukocyte-permissive sites in the postcapillary venules (Nourshargh et al., 2010) as well as the expression profile of recruited leukocytes. Specifically, because pericytes, together with endothelial cells, contribute to the generation of the venular basement membrane, the loose net-like coverage of pericytes creates a basement membrane that shows a discontinuous expression of extracellular matrix proteins (more porous), with gaps in coverage acting as gates for migrating leukocytes in a wide range of inflammatory conditions. As noted above, pericytes are efficient “signal integrators” that provide maturation signals for vascular growth and control immune cell transit from the blood into underlying tissues (Kolaczkowska and Kubes, 2013). In the bone marrow, NG2^+^ pericyte were shown to maintain the retention of hematopoietic stem cells (HSC) by secreting chemokines and growth factors (Stark et al., 2018; Nobre et al., 2021). On the other hand, in postcapillary venules NG2^–^ pericytes were shown to promote leukocyte transmigration and abluminal crawling (Stark et al., 2018). In the interstitial space NG2^+^α-SMA^–^ pericytes can guide leukocytes to a focus of inflammation.

Not far from pericytes and its local microenvironment, Cerebrospinal fluid (CSF) is circulating and being pumped along the brain perivascular pathway facilitated by aquaporin-4 (AQP4) water channels in the astrocytic endfeet. CSF homeostasis has been recently redefined by the discovery of the glymphatic system (Iliff et al., 2012). It was only very recently that Kipnis and colleagues (Louveau et al., 2015) discovered that the mouse dura possesses lymphatic structures that are critical for fluid homeostasis and immune surveillance. Notably, Bedussi et al. (2017) achieved global labeling of the perivascular system, provide evidence of fluorescent probe accumulation around and between smooth muscle pericytes and capillaries. Using the PDGF retention motif knockout mouse line, Pdgfb^*ret/ret*^, in which PDGF-B tethering to endothelial cells is lost, impairing its function, they showed that the development of glymphatic function was suppressed and AQP4 polarization at astrocytic endfeet was decreased (Munk et al., 2019). Recent studies have questioned the impacts of the light-dark cycle, brain temperature, and blood flow on the function of the glymphatic system—a process in which we hypothesize that pericytes might play a central homeostatic role (Cai et al., 2020; Hablitz et al., 2020). The interplay between cerebral blood flow, sleep stage, glymphatic function and pericyte biology clearly will require further investigation. Blood flow in the orbitofrontal cortex has been shown to fluctuate throughout the sleep-wake cycle (Braun et al., 1997; Massimini et al., 2004). It was further shown that Slow-Wave Sleep (SWS) and sleep spindles are inversely correlated with blood flow in the prefrontal cortex (Tüshaus et al., 2017). With their role in matching capillary blood flow to the heterogeneity of neuronal activity, the potential role of pericytes during sleep and/or the buildup of sleep pressure needs to be investigated. It has been also shown that CBF in the thalamus decreases drastically as a function of delta and spindle activity during SWS which possibly regulates the loss of consciousness during this state (Hofle et al., 1997). Interestingly, an endogenous memory reactivation process occurs during sleep that is temporally entrained by slow oscillation-spindle complexes (Schreiner et al., 2021) which raises the question of whether pericytes could play a role in memory consolidation during sleep by controlling cerebral blood flow, which could also exhibit a differential regional distribution.

### “Activated Pericytes” Send “Wake-Up” Calls for Tumor Cells, Stem Cells, and Neurons

50 years ago, Folkman et al. (1971) hypothesized that the switch of a tumor from a dormant state to malignancy is driven by the development of vasculature. Stable microvasculature was shown to constitute a “dormant niche,” whereas neoangiogenic sprouts initiate metastatic outgrowth (Ghajar et al., 2013; Weis and Cheresh, 2013). A multifaceted role of pericytes in various critical events of Glioblastoma Multiforme (GBM) initiation, establishment, maintenance, and progression has been proposed (Sattiraju and Mintz, 2019). Strikingly, GBM-activated pericytes were shown to support tumor growth via immunosuppression (Valdor et al., 2017; Sena et al., 2018). Taken together, an angiogenic switch or burst that is characterized by an imbalance between pro- and anti-angiogenic factors has the ability to alter the dormant state and trigger tumor invasion (Indraccolo et al., 2006; Risson et al., 2020).

The vasculature is crafted from a mosaic of cells from different sources (Bautch, 2011). As noted above, pericyte-endothelial interactions are hypothesized to be critical for maintaining pericyte properties and/or holding them in “suspended animation” (Bautch, 2011). During angiogenesis, mesenchymal stem cells are ‘captured’ by developing vessels and become pericytes while retaining nascent stem cell properties which can be later reactivated after injury (Feng et al., 2011). Due to pericyte’s perivascular position and their multilineage potential, they can be considered the *in situ* equivalent of bone marrow mesenchymal or perivascular stromal cells. The extent to which pericytes act as Mesenchymal Stem Cells (MSCs) during tissue growth and repair has long been a controversial and circular argument (Guimarães-Camboa et al., 2017). MSCs were shown to express genes that are also expressed by pericytes claiming a pericyte origin (Feng et al., 2011; Winkler et al., 2011). On the other hand, isolation and culture of pericytes from tissues, based on the expression of these MSC markers and properties have provided convincing evidence that pericytes may act as a source of MSCs (Feng et al., 2011).

The adult vasculature can shape a niche for organ-specific stem cells, provide a pool of pericytes contributing to the tissue-specific mesenchymal cells and the local adventitial progenitors (Bautch, 2011; Corselli et al., 2013). An interesting study showed that Hematopoietic Stem Cells (HPSC)-derived neural crest stem cells (NCSCs) can differentiate into mural cells that express pericyte markers (Stebbins et al., 2019). These pericyte-like cells were shown to self-assemble with endothelial cells to recapitulate BBB function and key pericyte-driven phenotypes in the brain microvasculature, including enhanced barrier properties and reduced transcytosis (Goldberg and Hirschi, 2009; Stebbins et al., 2019). Capillary pericytes in the forebrain and bone marrow share some commonalities, including their location near HPSCs and their NCSC derivation. Such a specialized perivascular microenvironment, with less permeable blood vessels, maintains hematopoietic stem cells in a low ROS state regulating their activation states (Birbrair et al., 2015; Itkin et al., 2016; Mangialardi et al., 2016; Girolamo et al., 2021), an observation that has a bearing on the previously mentioned critical role of ROS in pericyte biology in health and disease.

Pericytes can also rapidly relay information to neurons. An interesting study by Yu and colleagues (Duan et al., 2018) showed that specific PDGFRβ-expressing cell subtypes of the neurovascular unit rapidly release the chemokine CCL2 after systemic infection, leading to increased neural excitability. As noted above, pericytes are known to respond rapidly to neuronal activity to modulate capillary perfusion and hence OEF. What is also interesting about this interaction is the crosstalk between brain-wide mural cells and subcortical neurons. Perivascular sympathetic innervation of the microvasculature is a critical regulator of immune and stem cell trafficking and homeostasis. Upon entering the Virchow–Robin space, this sympathetic innervation disappears and is completely absent in vessels within the brain parenchyma (Chédotal and Hamel, 1990). Instead, parenchymal arterioles and cortical microvessels are innervated from within the brain tissue, an innervation considered ‘intrinsic’ (Cohen et al., 1997). These vessels receive nerve afferents from subcortical neurons, for example, from the brain stem opioidergic signaling (Cetas et al., 2009), locus coeruleus (Goadsby and Duckworth, 1989), Raphe nucleus, basal forebrain, and cortical interneurons that project to the perivascular space, thereby relaying information to brain-wide microvascular pericytes and SMCs (Cohen et al., 1997; Bekar et al., 2012; Toussay et al., 2013). This closed circuit of pericyte-neuronal crosstalk inside or outside of the CNS is believed to be central for regulating brain control of other organs (e.g., immune and circulatory systems) and their homeostasis.

## When Microvascular Transportation Engineers Go Missing

### The ‘Two-Hit’ Hypothesis of Cognitive Decline and Alzheimer’s Disease

**FIGURE 2:**
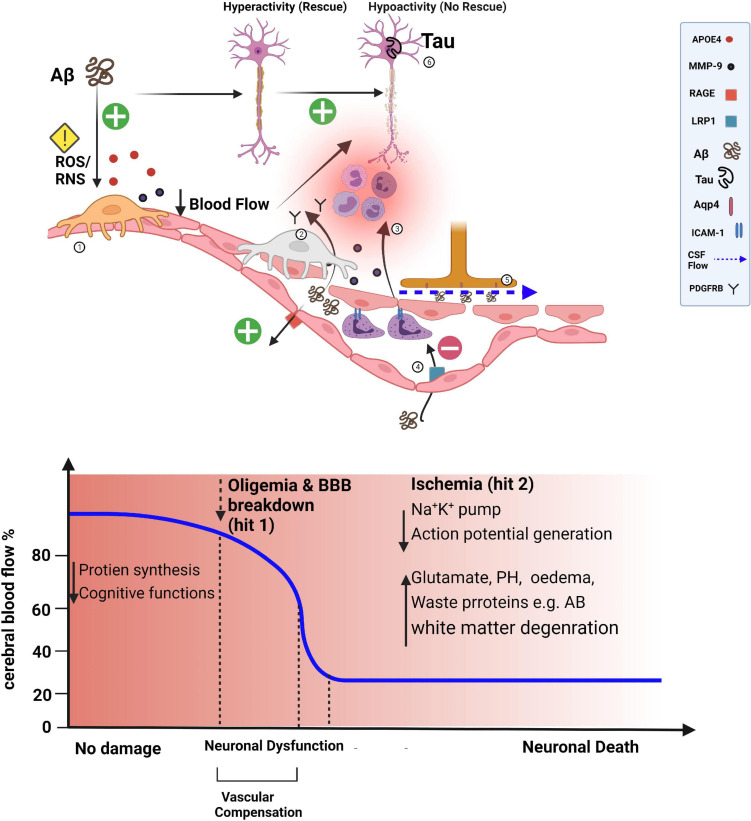
Losing transportation engineers of the microvasculature. The blood-brain barrier (BBB) influences brain levels of Aβ by regulating its entry into the brain via the receptor for advanced glycation end products (RAGE)-dependent transport and by controlling its clearance. Aβ can also lead to a pronounced capillary constriction through ROS/RNS in pericytes leading to an oligemia (hit 1). In *APOE4* high-risk people, secreted *APOE4* activates the release of MMP9 in pericytes disrupting endothelial tight junctions with a more influx of Aβ. The net result of processes that are pronounced (+) or diminished (−) in Alzheimer’s disease reflects an increased Aβ burden in the brain, which can further induce pericyte death (detected by higher soluble PDGFR-β in the cerebral spinal fluid), leading to BBB disruption and possibly contributing to perivascular deposition of Aβ aided by the dysfunctional glymphatic system (dashed blue arrow) (hit 2). Furthermore, upregulation of leukocyte adhesion molecules on endothelium causes increased attachment of leukocytes in the lumen of pericyte-deficient blood vessels and more invasion of the brain parenchyma. The combined presence of Aβ and tau pathology in the cortex can suppress neuronal activity and lead to axonal degeneration (exacerbated by brain ischemia). Suppression of Aβ or tau pathology alone is not effective in the rescue process.

The loss of ‘vascular resilience’ within the brain is a harbinger of the onset of age-related cognitive disorders (Arenaza-Urquijo and Vemuri, 2018). Chronic disease in the form of cardio-metabolic disorders, hypertension, BBB dysfunction, decreased Aβ clearance, sleep disruption, vascular oxidative stress, and reduced neurovascular coupling can all modulate vascular resilience (Grandner et al., 2016). A recent study (Perosa et al., 2020) proposed that vascular reserve is a marker of “brain resilience,” showing that the hippocampus has a mixed blood-delivery system and that the accompanying increase in reliability of the blood supply, as well as vascular reserve, protect against cognitive decline. In agreement with the selective vulnerability hypothesis of the hippocampus to damage and hypoxia, it was recently shown that diminished hippocampal neurovascular coupling contributes to the vulnerability of the hippocampus to damage in Alzheimer’s disease (Shaw et al., 2021). The interplay between vascular and neuronal oxidative stresses has been shown to precede and exacerbate Aß pathology (Massaad, 2011). The ‘two-hit vascular hypothesis’ of Alzheimer’s disease states proposed by Nelson et al. (2016b) posits that cerebrovascular damage (hit 1) is sufficient to initiate neuronal injury and that the resulting neurodegeneration leads to accumulation of Aβ in the brain (hit 2). Zhu et al. (2004) also proposed a two-hit hypothesis of Alzheimer’s disease, emphasizing the susceptibility of neurons to two independent insults: oxidative stress and cell cycle-related abnormalities. They further suggested that a common underlying mechanism could lead to this and other neurodegenerative diseases. The fact that neuronal health is intimately linked to the ability of the BBB to provide energy, clear waste, and protect neurons from circulating danger signals highlights the importance of preventing oxidative stress. We hypothesize that pericyte dysfunction could contribute to the two-hit model of Alzheimer’s disease by virtue of their essential roles in angiogenesis, BBB maintenance, and cerebral blood flow (Figure 2). A recent study showed that a decrease in cerebral blood flow in Alzheimer’s brains can initiate non-productive angiogenesis, leading to the disassembling of mature blood vessels into non-conducting tip cells around Aß plaques (Alvarez-Vergara et al., 2021).

Cerebrovascular disease is a strong predictor of cognitive decline in elderly populations. A characteristic feature of most Alzheimer’s disease patients is the presence of amyloid deposits in cerebral arteries, a pathological condition known as cerebral amyloid angiopathy (CAA). CAA impairs BBB function, increasing the risk of cerebral ischemia, microbleeds, and infections, all of which contribute to cognitive decline. Apolipoprotein E4 (*APOE*4), one of three polymorphic alleles (ε2, ε3, and ε4) of the *APOE* gene, is the most potent genetic risk factor for CAA and sporadic Alzheimer’s disease (Serrano-Pozo et al., 2021). A recent study (Blanchard et al., 2020) hypothesized that upregulation of *APOE4* in human pericytes underpins the pathogenic effects of *APOE4* in CAA. In aged *APOE4* knock-in mice expressing a humanized *APOE4* allele (hereafter, *APOE4* mice), cerebral vessel density is reduced and there is evidence of vascular atrophy. Even though the basement membrane normally thickens with age, the basement membrane of aged *APOE4* mice is thinner than that of aged *APOE2* or *APOE3* mice, an observation that might be explained by pericyte expression of *APOE4* and a decrease in collagen IV deposition. Also, the interaction of LRP1 (LDL receptor-related protein 1) with *APOE* is known to be reduced together with *APOE4*, further leading to a decrease in Aβ transport across the BBB (Henstridge et al., 2019).

The rate of cerebral spinal fluid turnover slows with healthy aging (Louveau et al., 2015). In aged mice, the glymphatic system is impaired due to loss of perivascular localization of the astrocytic endfoot water channel, AQP4, which is regulated at the transcriptional level by PDGFB signaling in the BBB (Munk et al., 2019). Sleep fragmentation, which is common with healthy aging, may disrupt perivascular movement, which is more active during slow-wave sleep (Nedergaard and Goldman, 2020). Reduced arterial compliance affects both vascular pulsations in the microvasculature (Seki et al., 2006; Kim et al., 2017) and glymphatic activity (Mestre et al., 2018) with otherwise healthy human aging. In addition, age-related decreases in the metabolic rate of brain cells or reductions in the number of brain cells reduce the production of metabolic free water as a result of pericyte-mediated chronic oligemia (Banks, 2016; Banks et al., 2021). Accordingly, pericyte failure might play a final common pathway to a broad range of neurodegenerative diseases.

There is thought to be a loss of pericytes with normal aging. As pericytes age, they lose contact with endothelial cells (Berthiaume et al., 2018). In mice, a 20% reduction in pericytes is sufficient to cause vascular damage without inducing neuronal damage (Nikolakopoulou et al., 2019). Pericytes are essential for BBB integrity, especially during times of acute cellular stress, and their absence causes severe changes in blood flow, including circulatory failure (Kisler et al., 2017; Henstridge et al., 2019; Banks et al., 2021). The soluble form of PDGFR shed by pericytes has been linked to BBB breakdown in humans, and levels of this protein in cerebral blood are associated with changes in cognitive status (Banks et al., 2021). Thus, BBB dysfunction may be caused by the age-related loss of pericytes. Pericytes can produce large quantities of *APOE*, various alleles of which influence cerebrovascular functions and determine the genetic risk for Alzheimer’s disease (Casey et al., 2015). In individuals with the *APOE4* allele, who are at high risk for Alzheimer’s disease, secreted *APOE*4 activates the release of matrix metalloproteinase-9 (MMP9) in pericytes, disrupting the junctions between endothelial cells and increasing the influx of Aβ into the brain (Figure 2; Ishii and Iadecola, 2020). The mechanisms underlying the age-related loss of pericytes, particularly that caused by oxidative DNA damage, are poorly understood and warrant further investigation.

The formation of Aβ fibrils and pericyte coverage reduction at the BBB were shown to have a positive feedback relationship that can synergically exacerbate vascular and parenchymal Aβ accumulation (Schultz et al., 2018). So, what are the mechanisms regulating this interaction? Pericytes are important regulators of transport systems, both in endothelial cells and within pericytes (Kisler et al., 2017; Henstridge et al., 2019). Continuous removal of Aß from the brain is essential for preventing the neurotoxic consequences of their accumulation from affecting vascular resilience. On the other hand, Aβ entry in the form of a free plasma-derived peptide can be facilitated by RAGE (receptor for advanced glycation end-products) or transported by immune cells. On the luminal membrane, Aβ binding to RAGE was shown to enhance the production of endothelin-1 leading vasoconstriction (Deane et al., 2003). LRP1 mediates the efflux of unbound Aβ as well as Aβ bound to *APOE*2, *APOE*3, and/or α2-macroglobulin from the parenchyma into the blood with the help of the ABCB1 transporter; notably, *APOE*4 inhibits this transport process (Nelson et al., 2016a). Aβ bound to clusterin is also transported through the BBB by LRP2 (Nelson et al., 2017). Taken together, Aβ is eliminated from the brain through enzymatic degradation as well as by removal via the BBB and glymphatic function (Deane et al., 2009; Iliff et al., 2012; Louveau et al., 2015).

In Alzheimer’s disease, pericyte degeneration or loss, as well as LRP1 downregulation, are the most common mechanisms that compromise the BBB (Alcendor, 2020). *APOE* genotype was shown to correlate with CBF (Wierenga et al., 2013). *APOE* was also shown to play an intrinsic role in pericyte mobility and maintenance of cerebrovascular function through a RhoA protein-mediated pathway (Casey et al., 2015), the same mechanism was attributed to a slow contraction in thin-stranded pericyte (Hartmann et al., 2021). A transcriptional study showed that the gene *ARHGAP42* encoding Rho GTPase activating protein-42 declines with age in both human and mouse BBB samples (He et al., 2016). This protein, which is abundant in pericytes, has been linked to blood pressure regulation, implying a role in vascular health. Rho GTPase signaling in pericytes has been shown to play critical roles in microvascular stabilization, maturation, and contractility (Kutcher et al., 2007).

In patients with Alzheimer’s disease and models of this disorder, the changes described above cause vessel regression, hypoperfusion, and Aβ accumulation resulting from the failure to clear Aβ via the BBB or through glymphatic function. Aβ has been shown to constrict pericytes through oxidative stress generation (Nortley et al., 2019). ROS generated in response to hypoperfusion and hypoxia mediated oxidative damage to the BBB, an effect that has been suggested to occur before neuronal degeneration and Aβ deposition in AD. *Meox2*^±^ mice, heterozygous for the transcription factor, mesenchyme homeobox 2, a gene expressed by the cardiovascular system that plays a major role in vascular differentiation (Soto et al., 2016), were shown to have normal pericyte coverage but exhibited concomitant hypoperfusion comparable to that found in pericyte-deficient mice (i.e., 45–50% reductions in capillary density and resting CBF, respectively) (Bell et al., 2010). Pericytes were also shown to contribute to rapid and localized proteolytic degradation of the BBB during cerebral ischemia (Zlokovic, 2011; Ishii and Iadecola, 2020)—a mechanism that was shown to play a role in the enhanced leakage of Aβ from the circulation into the brain and cognitive decline early in the course of Alzheimer’s disease (Cunnane et al., 2020). A better understanding of the fundamentals of cerebrovascular resilience is likely to lead to new insights into neurovascular function as well as novel diagnostic and therapeutic strategies.

### Do Pericytes Determine Stroke Outcome?

In stroke—the second leading cause of death worldwide—patients may experience paralysis, impaired speech, or vision due to acute ischemic events caused by thromboembolism, brain hemorrhages, and/or cardiac arrest (Moskowitz et al., 2010). Pericyte death affects several key aspects of vascular function, including capillary constriction, BBB function, and angiogenesis (Armulik et al., 2011). Several studies have shown that the “no-reflow” phenomenon that follows unblocking of the feeding artery after ischemic stroke causes a long-term reduction in cerebral blood flow (Vates et al., 2010; Cai et al., 2017). Pericytes are thought to play a significant role in this process. At the start of ischemia, inadequacies in blood and O_2_ delivery as well as mitochondrial dysfunction cause pericytes to constrict (Dalkara, 2019). One plausible explanation for this could be that, under hypoxic conditions, the absence of ATP needed to pump Ca^2+^ out of the cell leads to an increase in intracellular Ca^2+^ and activation of the contractile machinery, a condition that worsens during reperfusion after stroke (Hall et al., 2014). Alternatively, the sustained constricted state could reflect alterations in the release of vasoactive peptides (Gautam and Yao, 2018). Under ischemic conditions, when ATP levels are expected to be low, pericytes are vulnerable to damage. This raises the possibility that pericytes constrict capillaries at the start of a stroke and then remain in ‘rigor,’ causing the capillaries to remain too small to allow RBCs to pass through (Rustenhoven et al., 2017; Khennouf et al., 2018).

Since scavenging of superoxide and blocking nitric oxide (NO) production before reperfusion restores capillary patency, oxidative–nitrative stress has also been suggested as a cause of sustained pericyte constriction. Pericytes are very sensitive to the generation of ROS (Shah et al., 2013a). Another recent study showed that excessive ROS generation prevents endothelial cells from producing vasodilating mediators, resulting in the accumulation of opposing vasoconstricting peptides, which cause pericyte constriction (Rustenhoven et al., 2017). Computational modeling studies have shown that even minor changes in capillary constriction and tone following ischemia can increase the heterogeneity of capillary blood flow, reducing tissue oxygen extraction (Gonzales et al., 2020; Østergaard, 2020). As a result, even sparse or random pericyte constriction may contribute to the “no-reflow” phenomenon by not only reducing overall blood flow but also increasing the heterogeneity of capillary blood flow and oxygen extraction. The long-term effects of experimental ischemia on pericyte constriction and capillary blood flow may limit some of our current treatments for restoring perfusion, including rapid clot dissolution with tissue plasminogen activator (t-PA; Yemisci et al., 2009). This suggests that retaining normal pericyte function in conjunction with t-PA application could be worthwhile and that infusing antioxidants alongside t-PA could be a useful adjuvant therapy.

**FIGURE 3:**
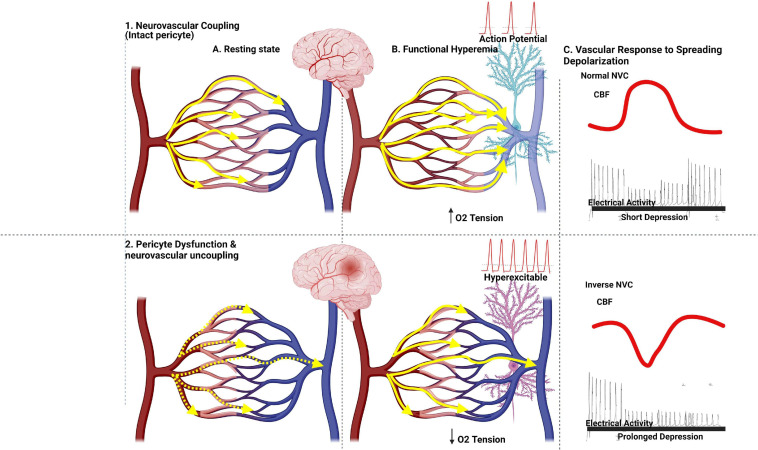
Capillary flow patterns controlled by pericytes govern the efficacy of oxygen extraction fraction in healthy and ischemic brains. Capillary flow patterns (yellow arrows) govern the efficacy of OEF values. Intravascular colors indicate blood saturation (red, more oxygenated; darker blue, more deoxygenated). In the resting state, RBCs’ velocities vary among capillaries, with little oxygen being extracted from blood. With normal neuronal activity, an increase in blood flow shortens capillary transit times and hence increases CTH-related functional shunting of oxygenated blood. With a more pronounced neuronal activity in a healthy brain, the response of the normal brain to spreading depolarization is accompanied by an increase in blood flow and a short depression in electrical activity. Bottom, pericyte loss and/or neurovascular uncoupling state associated by hyperexcitable neurons in an ischemic brain, an elevated CTTH, and failure of capillary flow patterns to homogenize during hyperemia hinder the redistribution of blood across the capillary bed and a decrease in blood flow in the capillary network, while less affected capillary paths act as functional shunts for oxygenated blood. The accompanying reduction in net oxygen supply reduces tissue oxygen tension as cells continue to use oxygen, increasing blood-tissue concentration gradients and net oxygen extraction. The response of the ischemic brain with dysfunctional pericytes to spreading depolarization is accompanied by a decrease in cerebral blood flow (ischemia) and prolonged depression in electrical activity.

After some ischemic and hemorrhagic strokes or traumatic brain injury (Dreier, 2011), a wave of membrane potential depolarization can propagate across the brain, a phenomenon referred to as cortical spreading depression that leads to changes in dendritic and synaptic architecture, depression in electrical activity, and altered vascular responses. Various noxious stimuli have been used experimentally to initiate spreading depolarization, including potassium, glutamate, status epilepticus, hypoxia, hypoglycemia, and ischemia (Ghoshal and Claassen, 2017). Cortical spreading depression causes a change in the tone of resistance vessels, resulting in either transient hyperperfusion in healthy tissue or extreme hypoperfusion (spreading ischemia) in tissue at risk for progressive damage, both of which lead to lesion progression and infarction (inverse neurovascular coupling) (Dreier, 2011; Østergaard et al., 2015). In a study by Khennouf et al. (2018) characterizing the effects of physiological stimuli and cortical spreading depression on penetrating arterioles and capillaries, the authors hypothesized that impaired brain hyperemia after spreading depolarization contributes to prolonged cortical dysfunction in patients and that preventing pericyte constriction could reduce the long-lasting decrease in blood flow after spreading depolarization (Grubb et al., 2020; Figure 3). It was further shown that peri-infarct depolarizations are triggered by a transient mismatch in supply and demand in the ischemic brain (Sugimoto et al., 2020). Interestingly, a recent study (Bornstädt et al., 2015) proposed that minimizing sensory stimulation and hypoxic or hypotensive transients in stroke reduced the incidence of spreading depolarization and its adverse outcomes. Dissecting the role of pericytes in the differential effects of waves of spreading depolarization in the healthy versus ischemic brain warrants further investigation.

### Role of Pericytes in COVID-19 Pathology and Recovery

**FIGURE 4:**
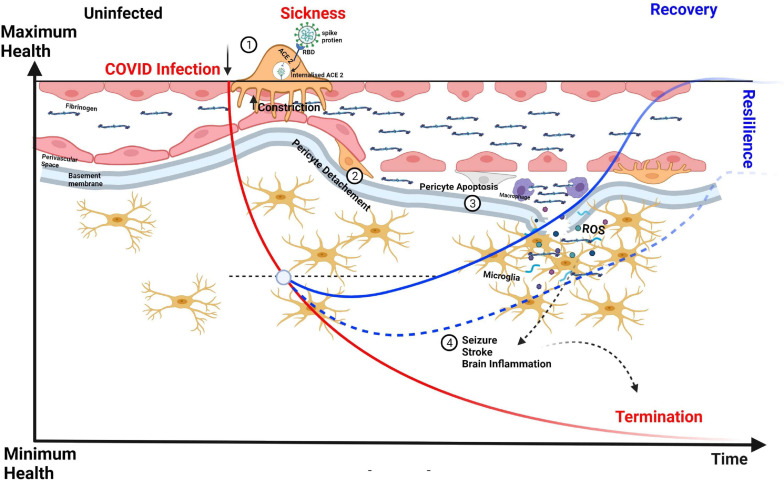
CNS capillary pericytes are a major target for SARS COV-2. The receptor for SARS-CoV-2 is the enzyme ACE2 which converts the vasoconstricting angiotensin II into vasodilating angiotensin. 1. The Spike protein of SARS-CoV-2 binds to ACE2 to trigger its endocytosis. 2. The neuropathological aspects of COVID-19 in post-mortem brain tissues show extravascular deposition of blood-borne products, e.g., fibrinogen and immune cells denoting a dysfunctional BBB leading associated with pericyte detachment and perhaps a more pronounced pericyte loss can lead to higher degrees of fibrinogen influx into the perivascular space and parenchyma with a pronounced formation oxidative stress leading to microglial activation, brain inflammation, and neuronal death. We hypothesize that vascular resilience and pericyte function is central in the recovery process, maintenance of BBB, and long-term effects of COVID-19.

To tackle the COVID-19 (Coronavirus Disease 2019) pandemic, research laboratories around the world have worked in overdrive to gain insights and develop treatments for the disease and its comorbidities. In brief, COVID-19 is caused by the severe acute respiratory syndrome coronavirus 2 (SARS-CoV-2), a strain of the virus that targets cells expressing angiotensin-converting enzyme 2 (ACE2; Østergaard, 2021). The spike protein of SARS-CoV-2 binds to ACE2, causing internalization of the SARS-CoV-2–ACE2 complex by endocytosis (Robinson et al., 2020; Figure 4). In the human lung, ACE2 is highly expressed in alveolar epithelial type II cells (Zou et al., 2020), and infection of these cells by SARS-CoV-2 leads to influenza-like symptoms ranging from mild respiratory distress to pneumonia, severe lung injury, organ failure, and death. Capillary pericytes also express ACE2, making them likely targets for SARS-CoV-2 infection (Chen et al., 2020). Consistent with this, patients with severe COVID-19 have been shown to exhibit a significant loss of capillary pericytes, as well as signs of extensive capillary angiogenesis in the lung (Ackermann et al., 2020). Notwithstanding the apparent increase in capillary healing, the absence of pericytes suggests an incomplete reparative process (Robinson et al., 2020).

Although respiratory symptoms dominate the spectrum of COVID-19 clinical manifestations, clinicians have reported extensive damage to other organs, including the brain and heart, where ACE2 receptors are also expressed (Xu and Lazartigues, 2020). Recent studies on neuropathological aspects of COVID-19 in post-mortem brain tissues have described extravascular deposition of blood-borne products, such as fibrinogen and immune cells, indicative of a dysfunctional BBB (Solomon et al., 2020; Lou et al., 2021) and microglial activation (Bouayed and Bohn, 2021). Upon exposure to hypoxia and oxidative stress, brain pericytes may die in rigor, disrupting blood perfusion and possibly also decreasing the delivery of O_2_ and nutrients to the tissue. Accordingly, this could be one of the mechanisms underlying the “brain fog” phenomenon described in many COVID-19 patients (Østergaard, 2020; Chilazi et al., 2021). A very recent interesting work used pericyte-containing organoids as an experimental model for COVID-19 infection showing that these novel “assembloids” support astrocytic maturation and confirmed the notion that pericytes are “replication hubs” helping virus spreading into the neuropil (Wang et al., 2021).

### Pericytes at the Crossroads of Neuroscience and Glioma Biology: From Mechanisms to Medicine

Glioblastoma multiforme (GBM), a type of malignant glioma, is a highly aggressive brain tumor that contains unique, self-renewing glioma stem cells (GSCs) with the ability to differentiate into vascular pericytes that support vessel function and tumor growth (Cheng et al., 2013). During GBM development, the highly vascularized tissue takes on an abnormal barrier appearance, resulting in the formation of a blood–tumor barrier (BTB; Arvanitis et al., 2020). The BTB exhibits a reduction in endothelial tight junctions and non-uniform pericyte vessel coverage, both of which compromise vascular integrity (Zhou et al., 2017; Deligne et al., 2020). GSCs lie within the perivascular hypoxic environment of tumors and are resistant to most medical interventions (Pietras et al., 2003). Because blood vessels in malignant brain tumors are leaky, dysfunctional, and immature, water and metabolic waste do not efficiently drain, leading to the buildup of interstitial and intracranial fluid pressure (Murayi and Chittiboina, 2016). Zhou et al. (2017) found that pericyte coverage is inversely related to the prognosis of glioblastoma patients treated with chemotherapy (Guerra et al., 2018), implying that targeting GSC-derived pericytes and the disrupted BTB may improve the delivery of chemotherapeutic agents.

Pericyte immunomodulation has been observed in a variety of conditions, including cancer (Turley et al., 2015; Liu et al., 2019). In brain pericytes activated by a gain-of-function mutation in PDGFR, the expression of immunoregulatory genes, including those involved in antigen presentation and interferon signaling, is increased (Turley et al., 2015). In a study of human malignant glioma—a brain tumor with extensive vascularization—researchers discovered a significant accumulation of pericytes in high-grade tumors that was negatively correlated with the presence of cytotoxic lymphocytes (Cheng et al., 2013; Valdor et al., 2019). These pericytes suppressed allogeneic and mitogen-activated T cell responses *ex vivo* by producing prostaglandin E2 (PGE_2_), transforming growth factor, and NO. Pericytes isolated from mouse melanoma and colon carcinoma models were shown to inhibit CD4^+^ T-cell proliferation, activation, and cytokine production. Furthermore, exposure to IL-6 enhanced pericyte immunoregulatory function, suggesting that tumor-derived factors may convert pericytes to a form that instructs the immune system in a manner beneficial to the tumor (Turley et al., 2015). Pericytes limit primary tumor cell intravasation through their stabilizing effects on vessels. When pericytes become depleted, the loosely assembled vessel wall is no longer an effective barrier against the dissemination of tumor cells; thus, a lack of pericytes around vessels has been linked to tumor metastasis (Xian et al., 2006). Notable in this context, a clinical trial of a PDGF-B blocker was halted because of excessive vascular leakage (Pietras et al., 2003; Hosaka et al., 2016; Liu et al., 2019). According to these studies, pericyte loss and the resulting prevention of vessel maturation can promote cancer, whereas other labs have reported that pericytes are co-opted by tumor cells at micro-metastatic sites through the release of angiogenic factors (Abramsson et al., 2003). Overall, more research into the benefits and risks of using PDGF blockade and pericyte-mediated control of the BTB could lead to new therapeutic interventions.

## Return of the Transportation Engineers

### Bench to Bedside: Microvascular Targets for Regenerative Medicine, Injection Therapies, and Deep Brain Stimulation in Neurodegenerative Disease

Protecting pericytes, and by extension, the BBB has been shown to halt, and in some cases reverse, the progression of many diseases. Inhibition of the MMP-9 pathway in pericytes was shown to maintain the cerebrovascular integrity required for normal neuronal function (Bell et al., 2012). Sleep also plays an integral role in maintaining the BBB and pericyte–capillary endothelial cell communication. Sleep deprivation increases the expression of low-grade inflammatory markers, including MMP-9, NF-κB, and the adenosine A2A receptor, and reduces expression of the markers of pericyte-endothelial cell interaction, PDGFR-β and connexin 43, leading to pericyte detachment and increase BBB permeability (Medina-Flores et al., 2020). Pharmacological inhibition of mitochondrial carbonic anhydrases (CAIs) has been shown to attenuate pericyte apoptosis and protect the BBB in the setting of diabetic-induced oxidative stress and pericyte loss (Shah et al., 2013b; Patrick et al., 2015), suggesting that CAIs can protect the BBB by reducing ROS generated by glucose metabolism, delaying age-related cerebral dysfunction. High glucose levels have been shown to cause pericyte apoptosis *in vitro*, an effect that was prevented by the antioxidant, ascorbic acid (May et al., 2014).

Pericytes, with their inherent actions on the microvasculature and multipotency, seem to act as ideal cells for microvascular regeneration (Corselli et al., 2010). Pericytes were shown to stimulate the host antioxidant system in a rodent model of Amyotrophic Lateral Sclerosis (Coatti et al., 2017). These results suggest that purified pericytes are ideal as an effective donor cell population for microvascular therapy and might also play a key role in restoring the antioxidative capacity of the tissue and preventing aging-related cognitive loss (Rustenhoven et al., 2017). Long-term exercise, caloric restriction, and inhibition of mTOR, the latter of which induces “vascular normalization” (Stoeltzing et al., 2006), might also help sustain pericyte function in aging mice (Van Skike and Galvan, 2018; Hadley et al., 2019). The re-introduction of pericytes through injection therapy following pericyte loss has been shown to improve BBB transport function and cerebral blood flow (Cathery et al., 2018). In one study, the right hemisphere of aged mice received an intracerebroventricular injection of pericytes derived from a mesenchymal stem cell (MSC) line. These cells were still alive 2 weeks later, and their pericyte properties were maintained, as evidenced by the facilitation of microcirculatory angiogenesis (Cheng et al., 2018). Pericytes can produce a number of growth factors. Advances in regenerative medicine and injection therapies will not only help with the re-introduction of pericytes but will also aid in replenishing these signaling molecules. In clinical trials, exogenously administered PDGF-BB was well tolerated in Parkinson’s disease patients (Paul et al., 2015). PDGF signaling is involved not only in the formation of blood vessels but also in their maintenance (Cheng et al., 2018). Glial cell line-derived neurotrophic factor (GDNF) released by pericytes was also shown to upregulate claudin-5 expression in endothelial cells, enhancing the barrier function. Pericyte-derived pleiotrophin (PTN), a secreted growth factor that is found at higher concentrations in pericytes, was shown to prevent neuronal loss and preserve blood flow. Taken together, these observations suggest that delivering PDGF-BB, PTN, and/or GDNF across the BBB could have therapeutic potential in the repair of an aging BBB (Cheng et al., 2018). Interestingly, Tachibana et al. (2018) reported that MSC-derived pericytes can reduce the Aβ burden in the brain, underscoring a promising cell-based therapeutic strategy against Alzheimer’s disease employing pericytes.

ROS signaling is critical for pericyte homeostasis in health and disease. The impact of ROS on the miRNA expression and the role of the ROS/miRNA feedback regulation can open new avenues for leveraging pericyte functions in health and disease. The cellular redox state is known to affect the sorting of miRNAs and can be manipulated to treat redox-associated disorders (for example, vasoconstriction in Alzheimer’s disease) (Nortley et al., 2019; Ruiz et al., 2021). Other applications of pericytes in regenerative medicine include facilitating wound healing and scar formation (Shechter and Schwartz, 2013). After a CNS injury, regeneration of lost tissue is limited, and the lesion is sealed with a scar. Using a spinal cord injury model, Frisén and colleagues (Göritz et al., 2011) showed that CNS damage caused a specific pericyte subtype to give rise to scar-forming stromal cells and further demonstrated that blocking this mechanism resulted in failure to seal the injured tissue. Pericytes and adventitial perivascular cells express MSC markers and display the capacity to differentiate. Importantly, adventitial cells can differentiate into pericyte-like cells (Crisan et al., 2012). Since adventitial cells are endowed with enhanced detoxifier and antioxidant systems (Iacobazzi et al., 2014), using purified perivascular adventitial cells instead of MSCs to reintroduce pericytes may bring greater benefits in regenerative medicine applications.

Deep brain stimulation (DBS), in which a surgically implanted device sends electrical signals to brain regions to modulate their activity, has been employed to treat multiple neurodegenerative diseases. In particular, DBS has been shown to improve microvascular integrity in the subthalamic nucleus in Parkinson’s disease (Pienaar et al., 2015). Endovascular delivery of current using electrode-bearing stents represents a potential alternative to conventional neurosurgical procedures (Shen, 2019), and multiple preclinical and clinical studies have shown that transcranial electrical stimulation has direct vascular effects (Zheng et al., 2011; Pulgar, 2015; Turner et al., 2021). These effects may influence neuronal activity, reversing the traditional view of the vasculature as secondary responders and instead of casting them as primary modulators and instructors of neuronal activity. However, further studies are warranted to elucidate the exact mechanisms underlying vascular responses to non-invasive electrical stimulation (Bahr-Hosseini and Bikson, 2021). Integration of previous approaches with the concept of pericytes as instructors of the CNS cellular communicome, i.e., “cellular communication factors including acute phase proteins, complement, cytokines, and chemokines” (Lucin and Wyss-Coray, 2009), should aid in the identification of interventions targeting early points in the course of neurodegenerative diseases.

### Synthetic Engineering of Capillary Networks: New Frontiers in *in vitro* Organoid Systems

Bioengineered blood vessels can incorporate living cells after being implanted in the human body, becoming blood-carrying, self-healing tubes that function as substitutes for human blood vessels (Kirkton et al., 2019). Pericyte loss leads to a breakdown and reorganization of the capillary network (Bergers and Song, 2005), and we propose that a bioengineered organoid system can efficiently provide a means of jumpstarting the angiogenic, or vascular replacement process. *In vitro* cell culture assays are typically based on non-physiological 2D cell cultures, which cannot reflect the complex architecture and cell-cell interactions as well as the blood perfusion (Kirkton et al., 2019). Until recently, *in vitro* and *ex vivo* models shared one major limitation, the lack of vascularization or vasculature-like perfusion (Redd et al., 2019). The invention of physiologically relevant *in vitro* models capable of mimicking human biology is critical. Organ-Chip technology offers a 3D engineered microscale system that mimics the cellular microenvironment by recreating multicellular architectures, tissue-tissue interfaces, mechanical forces, biophysical microenvironments, and perfusion (Vatine et al., 2019). Interestingly, merging organoid and organ-on-a-chip technology to generate complex multi-layer tissue models (Achberger et al., 2019). A very recent study used pericyte-containing organoids as an experimental model for COVID-19 infection showing that these novel “assembloids” support astrocytic maturation and SARS-CoV-2 entry and replication (Wang et al., 2021).

Recently, several efforts have focused on developing the techniques to vascularize organoids ranging from endothelial cell and pericyte co-cultures, *in vivo* transplantation, to integrating biomechanical cues and/or inducible genetic circuits. However, perfusable vasculature within the organoid has only been achieved via *in vivo* transplantation. It has been agreeable that engineering organoids with functional vasculature can be induced only by incorporating multiple factors that can work in a synergetic manner with the most suitable organoid generation protocols (Zhang et al., 2021). Organoids have recently been used to investigate the development and pathology of various tissue types (Lim et al., 2021). 3D brain organoids comprise multiple cell types and exhibit a cortical laminar organization, cellular compartmentalization, and organ-like functions (Wörsdörfer et al., 2020). Brain organoids lacking a vasculature—a critical component of all organs—can develop hypoxia during culture, hindering the normal development of neurons (Shirure et al., 2021). Accordingly, some studies have sought to generate vascularized organoids. Mansour and colleagues (Mansour et al., 2018) recently reported an *in vivo* organoid model, showing that human cerebral organoids transplanted into an adult mouse brain form a vascularized and functional brain organoid. It has also been demonstrated that engrafting cerebral organoids into a lesioned mouse cortex enhances survival and induces robust vascularization compared with transplantation of neural progenitor cells (Shi et al., 2020). Taken together, these observations support the long-term goal of regenerative medicine to create *in vitro* biomimetic organoids with complete vascular systems that restore normal function, an effort that involves replacing, engineering, and regenerating human vascular tissues (Hofer and Lutolf, 2021).

Recent groundbreaking research suggested that pluripotent stem cells can be used to create self-organizing 3D human blood vessel organoids. Endothelial cells and pericytes in these human blood vessel organoids were shown to self-assemble into capillary networks surrounded by a basement membrane (Wimmer et al., 2019b). Human blood vessel organoids transplanted into mice were shown to form a stable, perfused vascular tree, with arteries, arterioles, and venules (Wimmer et al., 2019a). In contrast to “self-organizing” vascular organoids, the pre-patterning approach often starts with constructing hydrogel scaffolds, either by utilizing various 3D printing methods and are then populated with ECs to form functional vascular networks. It was further shown that exposure of blood vessel organoids to hyperglycemia and inflammatory cytokines induced thickening of the vascular basement membrane (Grebenyuk and Ranga, 2019), setting the stage for the use of these organoids as a reliable model for therapeutics and diagnostics. Leveraging the power of individual 3D bio-fabrication technologies, or combinations of different techniques, to create vascularized organoids for studying pericyte biology should help answer major questions in neuroscience and vascular biology (Zhao and Chappell, 2019; Zhang et al., 2021).

## Conclusion

In this review, we have summarized the latest research on the important roles of pericytes in microvascular homeostasis, neuroinflammation, and neurodegeneration. In particular, we have highlighted the role of pericytes as ‘transportation engineers’ of the cerebral microvasculature, instructing and orchestrating (1) cerebral hemodynamics, (2) endothelial cell dynamics involved in blood vessel formation and maintenance, (3) immune cells and the perivascular microenvironment, and (4) stem cell niches and neurons. Pericytes in the brain and spinal cord are increasingly recognized as important mediators of neuroinflammation and regulators of barrier functions. They occupy a key perivascular niche and function as brain-wide signal integrators and instructors of the cellular communicome, making them critical regulators of the neurovascular unit. Understanding how pericytes contribute to brain inflammation, BBB dysfunction, and blood spinal cord barrier dysfunction can offer new insights into pharmacological strategies for treating a variety of neuroinflammatory disorders and injuries.

## Author Contributions

AE wrote the manuscript. YK designed the figures along with AE. AG reviewed and supervised of the manuscript writing and figure making processes. All authors contributed to the article and approved the submitted version.

## Conflict of Interest

The authors declare that the research was conducted in the absence of any commercial or financial relationships that could be construed as a potential conflict of interest.

## Publisher’s Note

All claims expressed in this article are solely those of the authors and do not necessarily represent those of their affiliated organizations, or those of the publisher, the editors and the reviewers. Any product that may be evaluated in this article, or claim that may be made by its manufacturer, is not guaranteed or endorsed by the publisher.

## References

[B1] Abramsson A. Lindblom P. Betsholtz C. (2003). Endothelial and nonendothelial sources of PDGF-B regulate pericyte recruitment and influence vascular pattern formation in tumors. *J. Clin. Investig.* 112 1142–1151. 10.1172/jci200318549 14561699 PMC213487

[B2] Achberger K. Probst C. Haderspeck J. Bolz S. Rogal J. Chuchuy J. (2019). Merging organoid and organ-on-a-chip technology to generate complex multi-layer tissue models in a human retina-on-a-chip platform. *Elife* 8:e46188. 10.7554/eLife.46188 PMC6777939 31451149

[B3] Ackermann M. Mentzer S. J. Kolb M. Jonigk D. (2020). Inflammation and intussusceptive angiogenesis in COVID-19: everything in and out of flow. *Eur. Respirat. J.* 56:2003147. 10.1183/13993003.03147-2020 33008942 PMC7530910

[B4] Alcendor D. J. (2020). Interactions between Amyloid-B Proteins and Human Brain Pericytes: Implications for the Pathobiology of Alzheimer’s Disease. *J. Clin. Med.* 9:1490. 10.3390/jcm9051490 32429102 PMC7290583

[B5] Alon R. Nourshargh S. (2013). Learning in motion: pericytes instruct migrating innate leukocytes. *Nat. Immunol.* 14 14–15. 10.1038/ni.2489 23238752

[B6] Alvarez-Vergara M. I. Rosales-Nieves A. E. March-Diaz R. Rodriguez-Perinan G. Lara-Ureña N. (2021). Non-productive angiogenesis disassembles Aß plaque-associated blood vessels. *Nat. Communicat.* 12:3098. 10.1038/s41467-021-23337-z PMC8149638 34035282

[B7] Amano M. Ito M. Kimura K. Fukata Y. Chihara K. Nakano T. (1996). Phosphorylation and activation of myosin by Rho-associated kinase (Rho-kinase). *J. Biol. Chem.* 271 20246–20249. 10.1074/jbc.271.34.20246 8702756

[B8] Angleys H. Jespersen S. N. Østergaard L. (2018). The effects of capillary transit time heterogeneity on the BOLD signal. *Hum. Brain Mapp.* 39 2329–2352. 10.1002/hbm.23991 29498762 PMC6866377

[B9] Arenaza-Urquijo E. M. Vemuri P. (2018). Resistance vs resilience to Alzheimer disease: Clarifying terminology for preclinical studies. *Neurology* 90 695–703. 10.1212/wnl.0000000000005303 29592885 PMC5894932

[B10] Armulik A. Abramsson A. Betsholtz C. (2005). Endothelial/Pericyte Interactions. *Circulat. Res.* 97 512–523. 10.1161/01.res.0000182903.16652.d7 16166562

[B11] Armulik A. Genové G. Betsholtz C. (2011). Pericytes: Developmental, Physiological, and Pathological Perspectives, Problems, and Promises. *Dev. Cell* 21 193–215. 10.1016/j.devcel.2011.07.001 21839917

[B12] Armulik A. Genové G. Mäe M. Nisancioglu M. H. Wallgard E. Niaudet C. (2010). Pericytes regulate the blood-brain barrier. *Nature* 468 557–561. 20944627 10.1038/nature09522

[B13] Arvanitis C. D. Ferraro G. B. Jain R. K. (2020). The blood-brain barrier and blood-tumour barrier in brain tumours and metastases. *Nat. Rev. Cancer* 20 26–41. 10.1038/s41568-019-0205-x 31601988 PMC8246629

[B14] Bahr-Hosseini M. Bikson M. (2021). Neurovascular-modulation: A review of primary vascular responses to transcranial electrical stimulation as a mechanism of action. *Brain Stimulat.* 14 837–847. 10.1016/j.brs.2021.04.015 33962079

[B15] Banks W. A. (2016). From blood–brain barrier to blood–brain interface: new opportunities for CNS drug delivery. *Nat. Rev. Drug Discov.* 15 275–292. 10.1038/nrd.2015.21 26794270

[B16] Banks W. A. Reed M. J. Logsdon A. F. Rhea E. M. Erickson M. A. (2021). Healthy aging and the blood–brain barrier. *Nat. Aging* 1 243–254. 10.1038/s43587-021-00043-5 34368785 PMC8340949

[B17] Bautch V. L. (2011). Stem cells and the vasculature. *Nat. Med.* 17 1437–1443. 22064433 10.1038/nm.2539

[B18] Bechmann I. Galea I. Perry V. H. (2007). What is the blood–brain barrier (not)? *Trends Immunol.* 28 5–11. 17140851 10.1016/j.it.2006.11.007

[B19] Bedussi B. Van Der Wel N. N. Vos J. Van Veen H. Siebes M. Vanbavel E. (2017). Paravascular channels, cisterns, and the subarachnoid space in the rat brain: A single compartment with preferential pathways. *J. Cereb. Blood Flow Metabol.* 37 1374–1385. 10.1177/0271678x16655550 27306753 PMC5453458

[B20] Bekar L. K. Wei H. S. Nedergaard M. (2012). The locus coeruleus-norepinephrine network optimizes coupling of cerebral blood volume with oxygen demand. *J. Cereb. Blood Flow Metabol.* 32 2135–2145. 10.1038/jcbfm.2012.115 22872230 PMC3519408

[B21] Bell R. D. Winkler E. A. Sagare A. P. Singh I. Larue B. Deane R. (2010). Pericytes control key neurovascular functions and neuronal phenotype in the adult brain and during brain aging. *Neuron* 68 409–427. 10.1016/j.neuron.2010.09.043 21040844 PMC3056408

[B22] Bell R. D. Winkler E. A. Singh I. Sagare A. P. Deane R. Wu Z. (2012). Apolipoprotein E controls cerebrovascular integrity via cyclophilin A. *Nature* 485 512–516. 10.1038/nature11087 22622580 PMC4047116

[B23] Bergers G. Song S. (2005). The role of pericytes in blood-vessel formation and maintenance. *Neuro Oncol.* 7 452–464. 10.1215/s1152851705000232 16212810 PMC1871727

[B24] Berthiaume A.-A. Hartmann D. A. Majesky M. W. Bhat N. R. Shih A. Y. (2018). Pericyte Structural Remodeling in Cerebrovascular Health and Homeostasis. *Front. Aging Neurosci.* 10:210–210. 10.3389/fnagi.2018.00210 30065645 PMC6057109

[B25] Billaud M. Donnenberg V. S. Ellis B. W. Meyer E. M. Donnenberg A. D. Hill J. C. (2017). Classification and Functional Characterization of Vasa Vasorum-Associated Perivascular Progenitor Cells in Human Aorta. *Stem Cell Rep.* 9 292–303. 10.1016/j.stemcr.2017.04.028 28552602 PMC5511043

[B26] Birbrair A. Zhang T. Wang Z.-M. Messi M. L. Mintz A. Delbono O. (2015). Pericytes at the intersection between tissue regeneration and pathology. *Clin. Sci.* 128 81–93. 10.1042/cs20140278 25236972 PMC4200531

[B27] Blanchard J. W. Bula M. Davila-Velderrain J. Akay L. A. Zhu L. Frank A. (2020). Reconstruction of the human blood–brain barrier in vitro reveals a pathogenic mechanism of APOE4 in pericytes. *Nat. Med.* 26 952–963. 10.1038/s41591-020-0886-4 32514169 PMC7704032

[B28] Bornstädt D. Houben T. Seidel J. Zheng Y. Dilekoz E. Qin T. (2015). Supply-demand mismatch transients in susceptible peri-infarct hot zones explain the origin of spreading injury depolarizations. *Neuron* 85 1117–1131. 10.1016/j.neuron.2015.02.007 25741731 PMC4351476

[B29] Bouayed J. Bohn T. (2021). The link between microglia and the severity of COVID-19: The “two-hit” hypothesis. *J. Med. Virol.* 93 4111–4113. 10.1002/jmv.26984 33788265 PMC8250886

[B30] Braun A. R. Balkin T. J. Wesenten N. J. Carson R. E. Varga M. Baldwin P. (1997). Regional cerebral blood flow throughout the sleep-wake cycle. An H2(15)O PET study. *Brain J. Neurol.* 120(Pt 7) 1173–1197. 10.1093/brain/120.7.1173 9236630

[B31] Cai W. Liu H. Zhao J. Chen L. Y. Chen J. Lu Z. (2017). Pericytes in Brain Injury and Repair After Ischemic Stroke. *Transl. Stroke Res.* 8 107–121. 10.1007/s12975-016-0504-4 27837475 PMC5350040

[B32] Cai X. Qiao J. Kulkarni P. Harding I. C. Ebong E. Ferris C. F. (2020). Imaging the effect of the circadian light-dark cycle on the glymphatic system in awake rats. *Proc. Natl. Acad. Sci.* 117 668–676. 10.1073/pnas.1914017117 31848247 PMC6955326

[B33] Casey C. S. Atagi Y. Yamazaki Y. Shinohara M. Tachibana M. Fu Y. (2015). Apolipoprotein E Inhibits Cerebrovascular Pericyte Mobility through a RhoA Protein-mediated Pathway. *J. Biol. Chem.* 290 14208–14217. 10.1074/jbc.m114.625251 25903128 PMC4447989

[B34] Cathery W. Faulkner A. Maselli D. Madeddu P. (2018). Concise Review: The Regenerative Journey of Pericytes Toward Clinical Translation. *Stem Cells* 36 1295–1310. 10.1002/stem.2846 29732653 PMC6175115

[B35] Cetas J. S. Lee D. R. Alkayed N. J. Wang R. Iliff J. J. Heinricher M. M. (2009). Brainstem control of cerebral blood flow and application to acute vasospasm following experimental subarachnoid hemorrhage. *Neuroscience* 163 719–729. 10.1016/j.neuroscience.2009.06.031 19539726 PMC2738929

[B36] Chédotal A. Hamel E. (1990). Serotonin-synthesizing nerve fibers in rat and cat cerebral arteries and arterioles: immunohistochemistry of tryptophan-5-hydroxylase. *Neurosci. Lett.* 116 269–274. 10.1016/0304-3940(90)90085-n 2243604

[B37] Chen L. Li X. Chen M. Feng Y. Xiong C. (2020). The ACE2 expression in human heart indicates new potential mechanism of heart injury among patients infected with SARS-CoV-2. *Cardiovasc. Res.* 116 1097–1100. 10.1093/cvr/cvaa078 32227090 PMC7184507

[B38] Chen Q. Fisher D. T. Clancy K. A. Gauguet J.-M. M. Wang W.-C. Unger E. (2006). Fever-range thermal stress promotes lymphocyte trafficking across high endothelial venules via an interleukin 6 trans-signaling mechanism. *Nat. Immunol.* 7 1299–1308. 10.1038/ni1406 17086187

[B39] Cheng J. Korte N. Nortley R. Sethi H. Tang Y. Attwell D. (2018). Targeting pericytes for therapeutic approaches to neurological disorders. *Acta Neuropathol.* 136 507–523. 10.1007/s00401-018-1893-0 30097696 PMC6132947

[B40] Cheng L. Huang Z. Zhou W. Wu Q. Donnola S. Liu J. K. (2013). Glioblastoma stem cells generate vascular pericytes to support vessel function and tumor growth. *Cell* 153 139–152. 10.1016/j.cell.2013.02.021 23540695 PMC3638263

[B41] Chilazi M. Duffy E. Y. Thakkar A. Michos E. D. (2021). COVID and Cardiovascular Disease: What We Know in 2021. *Curr. Atheroscler. Rep.* 23:37. 10.1007/s11883-021-00935-2 PMC8117457 33983522

[B42] Cleuren A. C. A. Van Der Ent M. A. Jiang H. Hunker K. L. Yee A. Siemieniak D. R. (2019). The in vivo endothelial cell translatome is highly heterogeneous across vascular beds. *Proc. Natl. Acad. Sci.* 116 23618–23624. 10.1073/pnas.1912409116 31712416 PMC6876253

[B43] Coatti G. C. Frangini M. Valadares M. C. Gomes J. P. Lima N. O. Cavaçana N. (2017). Pericytes Extend Survival of ALS SOD1 Mice and Induce the Expression of Antioxidant Enzymes in the Murine Model and in IPSCs Derived Neuronal Cells from an ALS Patient. *Stem Cell Rev. Rep.* 13 686–698. 10.1007/s12015-017-9752-2 28710685

[B44] Coelho-Santos V. Shih A. Y. (2020). Postnatal development of cerebrovascular structure and the neurogliovascular unit. Wiley Interdisciplinary Reviews. *Dev. Biol.* 9:e363. 10.1002/wdev.363 PMC7027551 31576670

[B45] Coelho-Santos V. Berthiaume A.-A. Ornelas S. Stuhlmann H. Shih A. Y. (2021). Imaging the construction of capillary networks in the neonatal mouse brain. *Proc. Natl. Acad. Sci.* 118:e2100866118. 10.1073/pnas.2100866118 34172585 PMC8256089

[B46] Cohen Z. Molinatti G. Hamel E. (1997). Astroglial and Vascular Interactions of Noradrenaline Terminals in the Rat Cerebral Cortex. *J. Cereb. Blood Flow Metabol.* 17 894–904. 10.1097/00004647-199708000-00008 9290587

[B47] Corselli M. Chen C.-W. Crisan M. Lazzari L. Péault B. (2010). Perivascular Ancestors of Adult Multipotent Stem Cells. *Arterioscler. Thromb. Vascul. Biol.* 30 1104–1109. 10.1161/atvbaha.109.191643 20453168

[B48] Corselli M. Chin C. J. Parekh C. Sahaghian A. Wang W. Ge S. (2013). Perivascular support of human hematopoietic stem/progenitor cells. *Blood* 121 2891–2901. 23412095 10.1182/blood-2012-08-451864 PMC3707421

[B49] Crisan M. Corselli M. Chen W. C. W. Péault B. (2012). Perivascular cells for regenerative medicine. *J. Cell. Mol. Med.* 16 2851–2860. 10.1111/j.1582-4934.2012.01617.x 22882758 PMC4393715

[B50] Cuevas P. Gutierrez-Diaz J. A. Reimers D. Dujovny M. Diaz F. G. Ausman J. I. (1984). Pericyte endothelial gap junctions in human cerebral capillaries. *Anat. Embryol.* 170 155–159. 10.1007/bf00319000 6517350

[B51] Cunnane S. C. Trushina E. Morland C. Prigione A. Casadesus G. Andrews Z. B. (2020). Brain energy rescue: an emerging therapeutic concept for neurodegenerative disorders of ageing. *Nat. Rev. Drug Discov.* 19 609–633. 10.1038/s41573-020-0072-x 32709961 PMC7948516

[B52] Dalkara T. (2019). Pericytes. *Stroke* 50 2985–2991. 31495330 10.1161/STROKEAHA.118.023590

[B53] Daneman R. Zhou L. Kebede A. A. Barres B. A. (2010). Pericytes are required for blood-brain barrier integrity during embryogenesis. *Nature* 468 562–566. 10.1038/nature09513 20944625 PMC3241506

[B54] Deane R. Bell R. D. Sagare A. Zlokovic B. V. (2009). Clearance of amyloid-beta peptide across the blood-brain barrier: implication for therapies in Alzheimer’s disease. *CNS Neurol. Disord. Drug Targets* 8 16–30. 10.2174/187152709787601867 19275634 PMC2872930

[B55] Deane R. Du Yan S. Submamaryan R. K. Larue B. Jovanovic S. Hogg E. (2003). RAGE mediates amyloid-β peptide transport across the blood-brain barrier and accumulation in brain. *Nat. Med.* 9 907–913. 10.1038/nm890 12808450

[B56] Deligne C. Hachani J. Duban-Deweer S. Meignan S. Leblond P. Carcaboso A. M. (2020). Development of a human in vitro blood–brain tumor barrier model of diffuse intrinsic pontine glioma to better understand the chemoresistance. *Fluids Barriers CNS* 17:37. 10.1186/s12987-020-00198-0 PMC7268424 32487241

[B57] DeNofrio D. Hoock T. C. Herman I. M. (1989). Functional sorting of actin isoforms in microvascular pericytes. *J. Cell Biol.* 109 191–202. 10.1083/jcb.109.1.191 2745546 PMC2115462

[B58] Dessalles C. A. Babataheri A. Barakat A. I. (2021). Pericyte mechanics and mechanobiology. *J. Cell Sci.* 134:jcs240226. 10.1242/jcs.240226 33753399

[B59] Dreier J. P. (2011). The role of spreading depression, spreading depolarization and spreading ischemia in neurological disease. *Nat. Med.* 17 439–447. 10.1038/nm.2333 21475241

[B60] Duan L. Zhang X.-D. Miao W.-Y. Sun Y.-J. Xiong G. Wu Q. (2018). PDGFRβ Cells Rapidly Relay Inflammatory Signal from the Circulatory System to Neurons via Chemokine CCL2. *Neuron* 100 183.e–200.e. 30269986 10.1016/j.neuron.2018.08.030

[B61] Eilken H. M. Diéguez-Hurtado R. Schmidt I. Nakayama M. Jeong H.-W. Arf H. (2017). Pericytes regulate VEGF-induced endothelial sprouting through VEGFR1. *Nat. Commun.* 8:1574. 10.1038/s41467-017-01738-3 PMC5691060 29146905

[B62] Evans S. S. Repasky E. A. Fisher D. T. (2015). Fever and the thermal regulation of immunity: the immune system feels the heat. *Nat. Rev. Immunol.* 15 335–349. 10.1038/nri3843 25976513 PMC4786079

[B63] Fabry Z. Fitzsimmons K. M. Herlein J. A. Moninger T. O. Dobbs M. B. Hart M. N. (1993). Product ion of the cytokines interleukin 1 and 6 by murine brain microvessel endothelium and smooth muscle pericytes. *J. Neuroimmunol.* 47 23–34. 10.1016/0165-5728(93)90281-3 8376546

[B64] Feng J. Mantesso A. De Bari C. Nishiyama A. Sharpe P. T. (2011). Dual origin of mesenchymal stem cells contributing to organ growth and repair. *Proc. Natl. Acad. Sci.* 108:6503. 10.1073/pnas.1015449108 21464310 PMC3081015

[B65] Fernández-Klett F. Offenhauser N. Dirnagl U. Priller J. Lindauer U. (2010). Pericytes in capillaries are contractile in vivo, but arterioles mediate functional hyperemia in the mouse brain. *Proc. Natl. Acad. Sci.* 107 22290–22295. 10.1073/pnas.1011321108 21135230 PMC3009761

[B66] Folkman J. Merler E. Abernathy C. Williams G. (1971). Isolation of a tumor factor responsible for angiogenesis. *J. Exp. Med.* 133 275–288. 10.1084/jem.133.2.275 4332371 PMC2138906

[B67] Gautam J. Yao Y. (2018). Roles of Pericytes in Stroke Pathogenesis. *Cell Transplant.* 27 1798–1808. 10.1177/0963689718768455 29845887 PMC6300777

[B68] Ghajar C. M. Peinado H. Mori H. Matei I. R. Evason K. J. Brazier H. (2013). The perivascular niche regulates breast tumour dormancy. *Nat. Cell Biol.* 15 807–817. 10.1038/ncb2767 23728425 PMC3826912

[B69] Ghoshal S. Claassen J. (2017). Spreading depolarization and acute ischaemia in subarachnoid haemorrhage: the role of mass depolarization waves. *Brain* 140 2527–2529. 10.1093/brain/awx226 28969392

[B70] Girolamo F. De Trizio I. Errede M. Longo G. D’amati A. Virgintino D. (2021). Neural crest cell-derived pericytes act as pro-angiogenic cells in human neocortex development and gliomas. *Fluids Barr. CNS* 18:14. 10.1186/s12987-021-00242-7 PMC7980348 33743764

[B71] Goadsby P. J. Duckworth J. W. (1989). Low frequency stimulation of the locus coeruleus reduces regional cerebral blood flow in the spinalized cat. *Brain Res.* 476 71–77. 10.1016/0006-8993(89)91537-0 2914215

[B72] Goldberg J. S. Hirschi K. K. (2009). Diverse roles of the vasculature within the neural stem cell niche. *Regenerat. Med.* 4 879–897. 10.2217/rme.09.61 19903006 PMC2836203

[B73] Gonzales A. L. Klug N. R. Moshkforoush A. Lee J. C. Lee F. K. Shui B. (2020). Contractile pericytes determine the direction of blood flow at capillary junctions. *Proc. Natl. Acad. Sci.* 117 27022–27033. 10.1073/pnas.1922755117 33051294 PMC7604512

[B74] Göritz C. Dias D. O. Tomilin N. Barbacid M. Shupliakov O. Frisén J. (2011). A Pericyte Origin of Spinal Cord Scar Tissue. *Science* 333 238–242. 10.1126/science.1203165 21737741

[B75] Grandner M. A. Alfonso-Miller P. Fernandez-Mendoza J. Shetty S. Shenoy S. Combs D. (2016). Sleep: important considerations for the prevention of cardiovascular disease. *Curr. Opin. Cardiol.* 31 551–565. 10.1097/hco.0000000000000324 27467177 PMC5056590

[B76] Grebenyuk S. Ranga A. (2019). Engineering Organoid Vascularization. *Front. Bioengine. Biotechnol.* 7:39. 10.3389/fbioe.2019.00039 30941347 PMC6433749

[B77] Groothuis D. R. Vavra M. W. Schlageter K. E. Kang E. W. Itskovich A. C. Hertzler S. (2007). Efflux of drugs and solutes from brain: the interactive roles of diffusional transcapillary transport, bulk flow and capillary transporters. *J. Cereb. Blood Flow Metab.* 27 43–56. 10.1038/sj.jcbfm.9600315 16639426

[B78] Grubb S. Cai C. Hald B. O. Khennouf L. Murmu R. P. Jensen A. G. K. (2020). Precapillary sphincters maintain perfusion in the cerebral cortex. *Nat. Commun.* 11:395. 10.1038/s41467-020-14330-z PMC6971292 31959752

[B79] Guerra D. A. P. Paiva A. E. Sena I. F. G. Azevedo P. O. Silva W. N. Mintz A. (2018). Targeting glioblastoma-derived pericytes improves chemotherapeutic outcome. *Angiogenesis* 21 667–675. 10.1007/s10456-018-9621-x 29761249 PMC6238207

[B80] Guimarães-Camboa N. Cattaneo P. Sun Y. Moore-Morris T. Gu Y. Dalton N. D. (2017). Pericytes of Multiple Organs Do Not Behave as Mesenchymal Stem Cells In Vivo. *Cell Stem Cell* 20 345.e–359.e. 28111199 10.1016/j.stem.2016.12.006 PMC5337131

[B81] Hablitz L. M. Plá V. Giannetto M. Vinitsky H. S. Stæger F. F. Metcalfe T. (2020). Circadian control of brain glymphatic and lymphatic fluid flow. *Nat. Commun.* 11:4411. 10.1038/s41467-020-18115-2 PMC7468152 32879313

[B82] Hadley G. Beard D. J. Couch Y. Neuhaus A. A. Adriaanse B. A. Deluca G. C. (2019). Rapamycin in ischemic stroke: Old drug, new tricks? *J. Cereb. Blood Flow Metabol.* 39 20–35. 10.1177/0271678x18807309 30334673 PMC6311672

[B83] Hall C. N. Reynell C. Gesslein B. Hamilton N. B. Mishra A. Sutherland B. A. (2014). Capillary pericytes regulate cerebral blood flow in health and disease. *Nature* 508 55–60. 10.1038/nature13165 24670647 PMC3976267

[B84] Hamilton N. B. Attwell D. Hall C. N. (2010). Pericyte-mediated regulation of capillary diameter: a component of neurovascular coupling in health and disease. *Front. Neuroenerget.* 2:5. 10.3389/fnene.2010.00005 20725515 PMC2912025

[B85] Hartmann D. A. Berthiaume A.-A. Grant R. I. Harrill S. A. Koski T. Tieu T. (2021). Brain capillary pericytes exert a substantial but slow influence on blood flow. *Nat. Neurosci.* 24 633–645. 10.1038/s41593-020-00793-2 33603231 PMC8102366

[B86] Hattori K. Dias S. Heissig B. Hackett N. R. Lyden D. Tateno M. (2001). Vascular endothelial growth factor and angiopoietin-1 stimulate postnatal hematopoiesis by recruitment of vasculogenic and hematopoietic stem cells. *J. Exp. Med.* 193 1005–1014. 10.1084/jem.193.9.1005 11342585 PMC2193424

[B87] He L. Vanlandewijck M. Raschperger E. Andaloussi Mäe M. Jung B. Lebouvier T. (2016). Analysis of the brain mural cell transcriptome. *Sci. Rep.* 6:35108. 10.1038/srep35108 PMC5057134 27725773

[B88] Henstridge C. M. Hyman B. T. Spires-Jones T. L. (2019). Beyond the neuron–cellular interactions early in Alzheimer disease pathogenesis. *Nat. Rev. Neurosci.* 20 94–108. 10.1038/s41583-018-0113-1 30643230 PMC6545070

[B89] Hill R. A. Tong L. Yuan P. Murikinati S. Gupta S. Grutzendler J. (2015). Regional Blood Flow in the Normal and Ischemic Brain Is Controlled by Arteriolar Smooth Muscle Cell Contractility and Not by Capillary Pericytes. *Neuron* 87 95–110. 10.1016/j.neuron.2015.06.001 26119027 PMC4487786

[B90] Hofer M. Lutolf M. P. (2021). Engineering organoids. *Nat. Rev. Mater.* 2021 1–19. 10.1038/s41578-021-00279-y PMC7893133 33623712

[B91] Hofle N. Paus T. Reutens D. Fiset P. Gotman J. Evans A. C. (1997). Regional cerebral blood flow changes as a function of delta and spindle activity during slow wave sleep in humans. *J. Neurosci.* 17 4800–4808. 10.1523/jneurosci.17-12-04800.1997 9169538 PMC6573353

[B92] Hørlyck S. Cai C. Helms H. C. C. Lauritzen M. Brodin B. (2021). ATP induces contraction of cultured brain capillary pericytes via activation of P2Y-type purinergic receptors. *Am. J. Physiol. Heart Circulat. Physiol.* 320 H699–H712. 10.1152/ajpheart.00560.2020 33306443

[B93] Hosaka K. Yang Y. Seki T. Fischer C. Dubey O. Fredlund E. (2016). Pericyte-fibroblast transition promotes tumor growth and metastasis. *Proc. Natl. Acad. Sci.* 113 E5618–E5627. 27608497 10.1073/pnas.1608384113 PMC5035870

[B94] Iacobazzi D. Mangialardi G. Gubernator M. Hofner M. Wielscher M. Vierlinger K. (2014). Increased Antioxidant Defense Mechanism in Human Adventitia-Derived Progenitor Cells Is Associated with Therapeutic Benefit in Ischemia. *Antioxid. Redox Signal.* 21 1591–1604. 10.1089/ars.2013.5404 24512058 PMC4174427

[B95] Iliff J. J. Wang M. Liao Y. Plogg B. A. Peng W. Gundersen G. A. (2012). A Paravascular Pathway Facilitates CSF Flow Through the Brain Parenchyma and the Clearance of Interstitial Solutes, Including Amyloid β. *Sci. Translat. Med.* 4 ra111–ra147. 10.1126/scitranslmed.3003748 PMC3551275 22896675

[B96] Indraccolo S. Favaro E. Amadori A. (2006). Dormant Tumors Awaken by a Short-Term Angiogenic Burst: The Spike Hypothesis. *Cell Cycle* 5 1751–1755. 10.4161/cc.5.16.2985 16861908

[B97] Institoris A. Gordon G. R. (2021). A tense relationship between capillaries and pericytes. *Nat. Neurosci.* 24 615–617. 10.1038/s41593-021-00853-1 33883740

[B98] Ishii M. Iadecola C. (2020). Risk factor for Alzheimer’s disease breaks the blood–brain barrier. *Nature* 581 31–32. 10.1038/d41586-020-01152-8 32350425 PMC8018585

[B99] Itkin T. Gur-Cohen S. Spencer J. A. Schajnovitz A. Ramasamy S. K. Kusumbe A. P. (2016). Distinct bone marrow blood vessels differentially regulate haematopoiesis. *Nature* 532 323–328. 10.1038/nature17624 27074509 PMC6450701

[B100] Jespersen S. N. Østergaard L. (2012). The roles of cerebral blood flow, capillary transit time heterogeneity, and oxygen tension in brain oxygenation and metabolism. *J. Cereb. Blood Flow Metabol.* 32 264–277. 10.1038/jcbfm.2011.153 22044867 PMC3272609

[B101] Kang T.-Y. Bocci F. Jolly M. K. Levine H. Onuchic J. N. Levchenko A. (2019). Pericytes enable effective angiogenesis in the presence of proinflammatory signals. *Proc. Natl. Acad. Sci.* 116 23551–23561. 10.1073/pnas.1913373116 31685607 PMC6876202

[B102] Khennouf L. Gesslein B. Brazhe A. Octeau J. C. Kutuzov N. Khakh B. S. (2018). Active role of capillary pericytes during stimulation-induced activity and spreading depolarization. *Brain* 141 2032–2046. 10.1093/brain/awy143 30053174 PMC6022680

[B103] Kim M. O. Li Y. Wei F. Wang J. O’rourke M. F. Adji A. (2017). Normal cerebral vascular pulsations in humans: changes with age and implications for microvascular disease. *J. Hypert.* 35 2245–2256. 10.1097/hjh.0000000000001459 28692445

[B104] Kirkton R. D. Santiago-Maysonet M. Lawson J. H. Tente W. E. Dahl S. L. M. Niklason L. E. (2019). Bioengineered human acellular vessels recellularize and evolve into living blood vessels after human implantation. *Sci. Translat. Med.* 11:eaau6934. 10.1126/scitranslmed.aau6934 30918113 PMC7557107

[B105] Kisler K. Nelson A. R. Montagne A. Zlokovic B. V. (2017). Cerebral blood flow regulation and neurovascular dysfunction in Alzheimer disease. *Nat. Rev. Neurosci.* 18 419–434. 10.1038/nrn.2017.48 28515434 PMC5759779

[B106] Kolaczkowska E. Kubes P. (2013). Neutrophil recruitment and function in health and inflammation. *Nat. Rev. Immunol.* 13 159–175. 10.1038/nri3399 23435331

[B107] Kovacs-Oller T. Ivanova E. Bianchimano P. Sagdullaev B. T. (2020). The pericyte connectome: spatial precision of neurovascular coupling is driven by selective connectivity maps of pericytes and endothelial cells and is disrupted in diabetes. *Cell Discov.* 6:39. 10.1038/s41421-020-0180-0 PMC7296038 32566247

[B108] Kutcher M. E. Kolyada A. Y. Surks H. K. Herman I. M. (2007). Pericyte Rho GTPase mediates both pericyte contractile phenotype and capillary endothelial growth state. *Am. J. Pathol.* 171 693–701. 10.2353/ajpath.2007.070102 17556591 PMC1934521

[B109] Lim J. Ching H. Yoon J.-K. Jeon N. L. Kim Y. (2021). Microvascularized tumor organoids-on-chips: advancing preclinical drug screening with pathophysiological relevance. *Nano Converg.* 8:12. 10.1186/s40580-021-00261-y PMC8042002 33846849

[B110] Lindskog H. Athley E. Larsson E. Lundin S. Hellström M. Lindahl P. (2006). New insights to vascular smooth muscle cell and pericyte differentiation of mouse embryonic stem cells in vitro. *Arterioscler. Thromb. Vasc. Biol.* 26 1457–1464. 10.1161/01.atv.0000222925.49817.17 16627807

[B111] Liu Z. Wang Y. Huang Y. Kim B. Y. S. Shan H. Wu D. (2019). Tumor Vasculatures: A New Target for Cancer Immunotherapy. *Trends Pharmacol. Sci.* 40 613–623. 10.1016/j.tips.2019.07.001 31331639 PMC7925217

[B112] Lou J. J. Movassaghi M. Gordy D. Olson M. G. Zhang T. Khurana M. S. (2021). Neuropathology of COVID-19 (neuro-COVID): clinicopathological update. *Free Neuropathol.* 2:2. 10.17879/freeneuropathology-2021-2993 PMC7861505 33554218

[B113] Louveau A. Smirnov I. Keyes T. J. Eccles J. D. Rouhani S. J. Peske J. D. (2015). Structural and functional features of central nervous system lymphatic vessels. *Nature* 523 337–341. 10.1038/nature14432 26030524 PMC4506234

[B114] Lucin K. M. Wyss-Coray T. (2009). Immune activation in brain aging and neurodegeneration: too much or too little? *Neuron* 64 110–122. 10.1016/j.neuron.2009.08.039 19840553 PMC2834890

[B115] Mangialardi G. Cordaro A. Madeddu P. (2016). The bone marrow pericyte: an orchestrator of vascular niche. *Regenerat. Med.* 11 883–895. 10.2217/rme-2016-0121 27885901 PMC5677781

[B116] Mansour A. A. Gonçalves J. T. Bloyd C. W. Li H. Fernandes S. Quang D. (2018). An in vivo model of functional and vascularized human brain organoids. *Nat. Biotechnol.* 36 432–441. 10.1038/nbt.4127 29658944 PMC6331203

[B117] Massaad C. A. (2011). Neuronal and vascular oxidative stress in Alzheimer’s disease. *Curr. Neuropharmacol.* 9 662–673. 10.2174/157015911798376244 22654724 PMC3263460

[B118] Massimini M. Huber R. Ferrarelli F. Hill S. Tononi G. (2004). The sleep slow oscillation as a traveling wave. *J. Neurosci.* 24 6862–6870. 10.1523/jneurosci.1318-04.2004 15295020 PMC6729597

[B119] Mastorakos P. McGavern D. (2019). The anatomy and immunology of vasculature in the central nervous system. *Sci. Immunol.* 4:eaav0492. 10.1126/sciimmunol.aav0492 31300479 PMC6816468

[B120] Matsumoto J. Dohgu S. Takata F. Machida T. Bölükbaşi Hatip F. F. Hatip-Al-Khatib I. (2018). TNF-α-sensitive brain pericytes activate microglia by releasing IL-6 through cooperation between IκB-NFκB and JAK-STAT3 pathways. *Brain Res.* 1692 34–44. 10.1016/j.brainres.2018.04.023 29702085

[B121] May J. M. Jayagopal A. Qu Z. C. Parker W. H. (2014). Ascorbic acid prevents high glucose-induced apoptosis in human brain pericytes. *Biochem. Biophys. Res. Commun.* 452 112–117. 10.1016/j.bbrc.2014.08.057 25152398 PMC4167575

[B122] Medina-Flores F. Hurtado-Alvarado G. Contis-Montes, De Oca A. Lopez-Cervantes S. P. Konigsberg M. (2020). Sleep loss disrupts pericyte-brain endothelial cell interactions impairing blood-brain barrier function. *Brain Behav. Immun.* 89 118–132. 10.1016/j.bbi.2020.05.077 32485292

[B123] Mestre H. Tithof J. Du T. Song W. Peng W. Sweeney A. M. (2018). Flow of cerebrospinal fluid is driven by arterial pulsations and is reduced in hypertension. *Nat. Commun.* 9:4878. 10.1038/s41467-018-07318-3 PMC6242982 30451853

[B124] Mintun M. A. Lundstrom B. N. Snyder A. Z. Vlassenko A. G. Shulman G. L. Raichle M. E. (2001). Blood flow and oxygen delivery to human brain during functional activity: theoretical modeling and experimental data. *Proc. Natl. Acad. Sci.* 98 6859–6864. 10.1073/pnas.111164398 11381119 PMC34443

[B125] Moskowitz M. A. Lo E. H. Iadecola C. (2010). The science of stroke: mechanisms in search of treatments. *Neuron* 67 181–198. 10.1016/j.neuron.2010.07.002 20670828 PMC2957363

[B126] Munk A. S. Wang W. Bèchet N. B. Eltanahy A. M. Cheng A. X. Sigurdsson B. (2019). PDGF-B Is Required for Development of the Glymphatic System. *Cell Rep.* 26 2955.e–2969.e. 30865886 10.1016/j.celrep.2019.02.050 PMC6447074

[B127] Murayi R. Chittiboina P. (2016). Glucocorticoids in the management of peritumoral brain edema: a review of molecular mechanisms. *Child’s Nervous Syst.* 32 2293–2302. 10.1007/s00381-016-3240-x 27613642 PMC5136308

[B128] Nedergaard M. Goldman S. A. (2020). Glymphatic failure as a final common pathway to dementia. *Science* 370 50–56. 10.1126/science.abb8739 33004510 PMC8186542

[B129] Nehls V. Drenckhahn D. (1993). The versatility of microvascular pericytes: from mesenchyme to smooth muscle? *Histochemistry* 99 1–12. 10.1007/bf00268014 8468190

[B130] Nelson A. R. Sagare A. P. Zlokovic B. V. (2016a). “Chapter 9 - Blood–Brain Barrier Transport of Alzheimer’s Amyloid β-Peptide,” in *Developing Therapeutics for Alzheimer’s Disease*, ed. Wolfe M. S. (Boston: Academic Press), 251–270. 10.1016/b978-0-12-802173-6.00009-5

[B131] Nelson A. R. Sagare A. P. Zlokovic B. V. (2017). Role of clusterin in the brain vascular clearance of amyloid-β. *Proc. Natl. Acad. Sci.* 114 8681–8682. 10.1073/pnas.1711357114 28765369 PMC5565473

[B132] Nelson A. R. Sweeney M. D. Sagare A. P. Zlokovic B. V. (2016b). Neurovascular dysfunction and neurodegeneration in dementia and Alzheimer’s disease. *Biochim. Biophys. Acta* 1862 887–900. 10.1016/j.bbadis.2015.12.016 26705676 PMC4821735

[B133] Nikolakopoulou A. M. Montagne A. Kisler K. Dai Z. Wang Y. Huuskonen M. T. (2019). Pericyte loss leads to circulatory failure and pleiotrophin depletion causing neuron loss. *Nat. Neurosci.* 22 1089–1098. 10.1038/s41593-019-0434-z 31235908 PMC6668719

[B134] Stricker S. (1871). *Handbuch der Lehre von den Geweben des Menschen und der Thiere.* Leipzig: Engelmann.

[B135] Rouget C. (1873). *Memoire sur le developpement, la structure et les proprietes physiologiques des capillaires sanguins.* Frankfurt: Universitätsbibliothek Johann Christian Senckenberg.

[B136] Nobre A. R. Risson E. Singh D. K. Di Martino J. S. Cheung J. F. Wang J. (2021). Bone marrow NG2+/Nestin+ mesenchymal stem cells drive DTC dormancy via TGF-β2. *Nat. Cancer* 2 327–339. 10.1038/s43018-021-00179-8 PMC8730384 34993493

[B137] Noguera-Troise I. Daly C. Papadopoulos N. J. Coetzee S. Boland P. Gale N. W. (2006). Blockade of Dll4 inhibits tumour growth by promoting non-productive angiogenesis. *Nature* 444 1032–1037. 10.1038/nature05355 17183313

[B138] Nortley R. Korte N. Izquierdo P. Hirunpattarasilp C. Mishra A. Jaunmuktane Z. (2019). Amyloid β oligomers constrict human capillaries in Alzheimer’s disease via signaling to pericytes. *Science* 365:eaav9518. 10.1126/science.aav9518 31221773 PMC6658218

[B139] Nourshargh S. Hordijk P. L. Sixt M. (2010). Breaching multiple barriers: leukocyte motility through venular walls and the interstitium. *Nat. Rev. Mol. Cell Biol.* 11 366–378. 10.1038/nrm2889 20414258

[B140] Østergaard L. (2020). Blood flow, capillary transit times, and tissue oxygenation: the centennial of capillary recruitment. *J. Appl. Physiol.* 129 1413–1421. 10.1152/japplphysiol.00537.2020 33031017

[B141] Østergaard L. (2021). SARS CoV-2 related microvascular damage and symptoms during and after COVID-19: Consequences of capillary transit-time changes, tissue hypoxia and inflammation. *Physiol. Rep.* 9:e14726. 10.14814/phy2.14726 PMC7849453 33523608

[B142] Østergaard L. Dreier Jens P. Hadjikhani N. Jespersen Sune N. Dirnagl U. Dalkara T. (2015). Neurovascular Coupling During Cortical Spreading Depolarization and –Depression. *Stroke* 46 1392–1401. 10.1161/strokeaha.114.008077 25882051

[B143] Patrick P. Price T. O. Diogo A. L. Sheibani N. Banks W. A. Shah G. N. (2015). Topiramate Protects Pericytes from Glucotoxicity: Role for Mitochondrial CA VA in Cerebromicrovascular Disease in Diabetes. *J. Endocrinol. Diabet.* 2:123. 10.15226/2374-6890/2/2/00123 PMC4495916 26167540

[B144] Paul G. Zachrisson O. Varrone A. Almqvist P. Jerling M. Lind G. (2015). Safety and tolerability of intracerebroventricular PDGF-BB in Parkinson’s disease patients. *J. Clin. Invest.* 125 1339–1346. 10.1172/jci79635 25689258 PMC4362250

[B145] Peppiatt C. M. Howarth C. Mobbs P. Attwell D. (2006). Bidirectional control of CNS capillary diameter by pericytes. *Nature* 443 700–704. 10.1038/nature05193 17036005 PMC1761848

[B146] Pereira F. A. Qiu Y. Zhou G. Tsai M. J. Tsai S. Y. (1999). The orphan nuclear receptor COUP-TFII is required for angiogenesis and heart development. *Genes Dev.* 13 1037–1049. 10.1101/gad.13.8.1037 10215630 PMC316637

[B147] Perosa V. Priester A. Ziegler G. Cardenas-Blanco A. Dobisch L. Spallazzi M. (2020). Hippocampal vascular reserve associated with cognitive performance and hippocampal volume. *Brain* 143 622–634. 10.1093/brain/awz383 31994699 PMC7009470

[B148] Pienaar I. S. Lee C. H. Elson J. L. Mcguinness L. Gentleman S. M. Kalaria R. N. (2015). Deep-brain stimulation associates with improved microvascular integrity in the subthalamic nucleus in Parkinson’s disease. *Neurobiol. Dis.* 74 392–405. 10.1016/j.nbd.2014.12.006 25533682

[B149] Pietras K. Sjöblom T. Rubin K. Heldin C.-H. Östman A. (2003). PDGF receptors as cancer drug targets. *Cancer Cell* 3 439–443. 10.1016/s1535-6108(03)00089-8 12781361

[B150] Proebstl D. Voisin M. B. Woodfin A. Whiteford J. D’acquisto F. Jones G. E. (2012). Pericytes support neutrophil subendothelial cell crawling and breaching of venular walls in vivo. *J. Exp. Med.* 209 1219–1234. 10.1084/jem.20111622 22615129 PMC3371725

[B151] Pulgar V. M. (2015). Direct electric stimulation to increase cerebrovascular function. *Front. Syst. Neurosci.* 9:54–54. 10.3389/fnsys.2015.00054 25870543 PMC4378276

[B152] Qin J. Chen X. Xie X. Tsai M.-J. Tsai S. Y. (2010). COUP-TFII regulates tumor growth and metastasis by modulating tumor angiogenesis. *Proc. Natl. Acad. Sci.* 107 3687–3692. 10.1073/pnas.0914619107 20133706 PMC2840495

[B153] Rafalski V. A. Merlini M. Akassoglou K. (2018). Pericytes: The Brain’s Very First Responders? *Neuron* 100 11–13. 10.1016/j.neuron.2018.09.033 30308164 PMC6701953

[B154] Redd M. A. Zeinstra N. Qin W. Wei W. Martinson A. Wang Y. (2019). Patterned human microvascular grafts enable rapid vascularization and increase perfusion in infarcted rat hearts. *Nat. Communicat.* 10:584. 10.1038/s41467-019-08388-7 PMC6362250 30718840

[B155] Risson E. Nobre A. R. Maguer-Satta V. Aguirre-Ghiso J. A. (2020). The current paradigm and challenges ahead for the dormancy of disseminated tumor cells. *Nat. Cancer* 1 672–680. 10.1038/s43018-020-0088-5 33681821 PMC7929485

[B156] Robinson F. A. Mihealsick R. P. Wagener B. M. Hanna P. Poston M. D. Efimov I. R. (2020). Role of angiotensin-converting enzyme 2 and pericytes in cardiac complications of COVID-19 infection. *Am. J. Physiol. Heart Circulat. Physiol.* 319 H1059–H1068. 10.1152/ajpheart.00681.2020 PMC7789968 33036546

[B157] Ruiz G. P. Camara H. Fazolini N. P. B. Mori M. A. (2021). Extracellular miRNAs in redox signaling: health, disease and potential therapies. *Free Radic. Biol. Med.* [Preprint]. 10.1016/j.freeradbiomed.2021.05.004 33965563

[B158] Rustenhoven J. Jansson D. Smyth L. C. Dragunow M. (2017). Brain Pericytes As Mediators of Neuroinflammation. *Trends Pharmacol. Sci.* 38 291–304. 10.1016/j.tips.2016.12.001 28017362

[B159] Sattiraju A. Mintz A. (2019). “Pericytes in Glioblastomas: Multifaceted Role Within Tumor Microenvironments and Potential for Therapeutic Interventions,” in *Pericyte Biology in Disease*, ed. Birbrair A. (Cham: Springer International Publishing), 65–91. 10.1007/978-3-030-16908-4_2 PMC7322693 31147872

[B160] Schmid F. Barrett M. J. P. Obrist D. Weber B. Jenny P. (2019). Red blood cells stabilize flow in brain microvascular networks. *PLoS Computat. Biol.* 15:e1007231–e1007231. 10.1371/journal.pcbi.1007231 31469820 PMC6750893

[B161] Schmid F. Tsai P. S. Kleinfeld D. Jenny P. Weber B. (2017). Depth-dependent flow and pressure characteristics in cortical microvascular networks. *PLoS Comput. Biol.* 13:e1005392. 10.1371/journal.pcbi.1005392 28196095 PMC5347440

[B162] Schreiner T. Petzka M. Staudigl T. Staresina B. P. (2021). Endogenous memory reactivation during sleep in humans is clocked by slow oscillation-spindle complexes. *Nat. Communicat.* 12 1–10. 10.1038/s41467-021-23520-2 PMC8149676 34035303

[B163] Schultz N. Brännström K. Byman E. Moussaud S. Nielsen H. M. Netherlands Brain B. (2018). Amyloid-beta 1-40 is associated with alterations in NG2+ pericyte population ex vivo and in vitro. *Aging Cell* 17 e12728–e12728. 29453790 10.1111/acel.12728 PMC5946076

[B164] Seki J. Satomura Y. Ooi Y. Yanagida T. Seiyama A. (2006). Velocity profiles in the rat cerebral microvessels measured by optical coherence tomography. *Clin. Hemorheol. Microcirc.* 34 233–239. 16543642

[B165] Sena I. F. G. Paiva A. E. Prazeres P. H. D. M. Azevedo P. O. Lousado L. Bhutia S. K. (2018). Glioblastoma-activated pericytes support tumor growth via immunosuppression. *Cancer Med.* 7 1232–1239. 10.1002/cam4.1375 29479841 PMC5911609

[B166] Serrano-Pozo A. Das S. Hyman B. T. (2021). APOE and Alzheimer’s disease: advances in genetics, pathophysiology, and therapeutic approaches. *Lancet Neurol.* 20 68–80. 10.1016/s1474-4422(20)30412-9 33340485 PMC8096522

[B167] Shah G. N. Morofuji Y. Banks W. A. Price T. O. (2013a). High glucose-induced mitochondrial respiration and reactive oxygen species in mouse cerebral pericytes is reversed by pharmacological inhibition of mitochondrial carbonic anhydrases: Implications for cerebral microvascular disease in diabetes. *Biochem. Biophys. Res. Commun.* 440 354–358. 10.1016/j.bbrc.2013.09.086 24076121 PMC3875343

[B168] Shah G. N. Price T. O. Banks W. A. Morofuji Y. Kovac A. Ercal N. (2013b). Pharmacological inhibition of mitochondrial carbonic anhydrases protects mouse cerebral pericytes from high glucose-induced oxidative stress and apoptosis. *J. Pharmacol. Exp. Ther.* 344 637–645. 10.1124/jpet.112.201400 23249625 PMC3583505

[B169] Shaw K. Bell L. Boyd K. Grijseels D. M. Clarke D. Bonnar O. (2021). Neurovascular coupling and oxygenation are decreased in hippocampus compared to neocortex because of microvascular differences. *Nat. Commun.* 12:3190. 10.1038/s41467-021-23508-y PMC8160329 34045465

[B170] Shechter R. Schwartz M. (2013). CNS sterile injury: just another wound healing? *Trends Mol. Med.* 19 135–143. 10.1016/j.molmed.2012.11.007 23279948

[B171] Shen H. H. (2019). Core Concept: Can deep brain stimulation find success beyond Parkinson’s disease? *Proc. Natl. Acad. Sci.* 116 4764–4766. 10.1073/pnas.1900442116 30862742 PMC6421452

[B172] Shi Y. Sun L. Wang M. Liu J. Zhong S. Li R. (2020). Vascularized human cortical organoids (vOrganoids) model cortical development in vivo. *PLoS Biol.* 18:e3000705–e3000705. 10.1371/journal.pbio.3000705 32401820 PMC7250475

[B173] Shirure V. S. Hughes C. C. W. George S. C. (2021). Engineering Vascularized Organoid-on-a-Chip Models. *Annu. Rev. Biomed. Engine.* [Preprint]. 10.1146/annurev-bioeng-090120-094330 33756087

[B174] Shum M. S. Pasquier E. Po’uha S. T. O’neill G. M. Chaponnier C. Gunning P. W. (2011). gamma-Actin regulates cell migration and modulates the ROCK signaling pathway. *FASEB J.* 25 4423–4433. 10.1096/fj.11-185447 21908715

[B175] Silva A. C. Lee S. P. Iadecola C. Kim S. G. (2000). Early temporal characteristics of cerebral blood flow and deoxyhemoglobin changes during somatosensory stimulation. *J. Cereb. Blood Flow Metabol.* 20 201–206. 10.1097/00004647-200001000-00025 10616809

[B176] Solomon I. H. Normandin E. Bhattacharyya S. Mukerji S. S. Keller K. Ali A. S. (2020). Neuropathological Features of Covid-19. *N. Engl. J. Med.* 383 989–992. 32530583 10.1056/NEJMc2019373 PMC7304421

[B177] Soto I. Grabowska W. A. Onos K. D. Graham L. C. Jackson H. M. Simeone S. N. (2016). Meox2 haploinsufficiency increases neuronal cell loss in a mouse model of Alzheimer’s disease. *Neurobiol. Aging* 42 50–60. 10.1016/j.neurobiolaging.2016.02.025 27143421 PMC4878023

[B178] Stark K. Eckart A. Haidari S. Tirniceriu A. Lorenz M. Brühl M.-L. (2013). Capillary and arteriolar pericytes attract innate leukocytes exiting through venules and ‘instruct’ them with pattern-recognition and motility programs. *Nat. Immunol.* 14 41–51. 10.1038/ni.2477 23179077

[B179] Stark K. Pekayvaz K. Massberg S. (2018). Role of pericytes in vascular immunosurveillance. *Front. Biosci. Landm.* 23:767–781. 10.2741/4615 28930571

[B180] Stebbins M. J. Gastfriend B. D. Canfield S. G. Lee M.-S. Richards D. Faubion M. G. (2019). Human pluripotent stem cell-derived brain pericyte-like cells induce blood-brain barrier properties. *Sci. Adv.* 5:eaau7375. 10.1126/sciadv.aau7375 30891496 PMC6415958

[B181] Stefanovic B. Hutchinson E. Yakovleva V. Schram V. Russell J. T. Belluscio L. (2008). Functional reactivity of cerebral capillaries. *J. Cereb. Blood Flow Metab.* 28 961–972. 10.1038/sj.jcbfm.9600590 18059431 PMC3197804

[B182] Stoeltzing O. Meric-Bernstam F. Ellis L. M. (2006). Intracellular signaling in tumor and endothelial cells: The expected and, yet again, the unexpected. *Cancer Cell* 10 89–91. 10.1016/j.ccr.2006.07.013 16904605

[B183] Sugimoto K. Chung David Y. Böhm M. Fischer P. Takizawa T. Aslihan Aykan S. (2020). Peri-Infarct Hot-Zones Have Higher Susceptibility to Optogenetic Functional Activation-Induced Spreading Depolarizations. *Stroke* 51 2526–2535. 10.1161/strokeaha.120.029618 32640946 PMC7387208

[B184] Suri C. Jones P. F. Patan S. Bartunkova S. Maisonpierre P. C. Davis S. (1996). Requisite Role of Angiopoietin-1, a Ligand for the TIE2 Receptor, during Embryonic Angiogenesis. *Cell* 87 1171–1180. 10.1016/s0092-8674(00)81813-9 8980224

[B185] Sweeney M. D. Ayyadurai S. Zlokovic B. V. (2016). Pericytes of the neurovascular unit: key functions and signaling pathways. *Nat. Neurosci.* 19 771–783. 10.1038/nn.4288 27227366 PMC5745011

[B186] Tachibana M. Yamazaki Y. Liu C.-C. Bu G. Kanekiyo T. (2018). Pericyte implantation in the brain enhances cerebral blood flow and reduces amyloid-β pathology in amyloid model mice. *Exp. Neurol.* 300 13–21. 10.1016/j.expneurol.2017.10.023 29106980 PMC5745278

[B187] Takakura N. Watanabe T. Suenobu S. Yamada Y. Noda T. Ito Y. (2000). A Role for Hematopoietic Stem Cells in Promoting Angiogenesis. *Cell* 102 199–209. 10.1016/s0092-8674(00)00025-8 10943840

[B188] Thomsen M. S. Routhe L. J. Moos T. (2017). The vascular basement membrane in the healthy and pathological brain. *J. Cereb. Blood Flow Metabol.* 37 3300–3317. 10.1177/0271678x17722436 28753105 PMC5624399

[B189] Toussay X. Basu K. Lacoste B. Hamel E. (2013). Locus Coeruleus Stimulation Recruits a Broad Cortical Neuronal Network and Increases Cortical Perfusion. *J. Neurosci.* 33 3390–3401. 10.1523/jneurosci.3346-12.2013 23426667 PMC6619527

[B190] Tsai A. G. Johnson P. C. Intaglietta M. (2003). Oxygen Gradients in the Microcirculation. *Physiol. Rev.* 83 933–963. 10.1152/physrev.00034.2002 12843412

[B191] Turley S. J. Cremasco V. Astarita J. L. (2015). Immunological hallmarks of stromal cells in the tumour microenvironment. *Nat. Rev. Immunol.* 15 669–682. 10.1038/nri3902 26471778

[B192] Turner D. A. Degan S. Galeffi F. Schmidt S. Peterchev A. V. (2021). Rapid, Dose-Dependent Enhancement of Cerebral Blood Flow by transcranial AC Stimulation in Mouse. *Brain Stimulat.* 14 80–87. 10.1016/j.brs.2020.11.012 33217607 PMC7855527

[B193] Tüshaus L. Omlin X. Tuura R. O. G. Federspiel A. Luechinger R. Staempfli P. (2017). In human non-REM sleep, more slow-wave activity leads to less blood flow in the prefrontal cortex. *Sci. Rep.* 7:14993. 10.1038/s41598-017-12890-7 PMC5670199 29101338

[B194] Valdor R. García-Bernal D. Bueno C. Ródenas M. Moraleda J. M. Macian F. (2017). Glioblastoma progression is assisted by induction of immunosuppressive function of pericytes through interaction with tumor cells. *Oncotarget* 8 68614–68626. 10.18632/oncotarget.19804 28978142 PMC5620282

[B195] Valdor R. García-Bernal D. Riquelme D. Martinez C. M. Moraleda J. M. Cuervo A. M. (2019). Glioblastoma ablates pericytes antitumor immune function through aberrant up-regulation of chaperone-mediated autophagy. *Proc. Natl. Acad. Sci.* 116 20655–20665. 10.1073/pnas.1903542116 31548426 PMC6789971

[B196] Van Skike C. E. Galvan V. (2018). A Perfect sTORm: The Role of the Mammalian Target of Rapamycin (mTOR) in Cerebrovascular Dysfunction of Alzheimer’s Disease: A Mini-Review. *Gerontology* 64 205–211. 10.1159/000485381 29320772 PMC5876078

[B197] Vates G. E. Takano T. Zlokovic B. Nedergaard M. (2010). Pericyte constriction after stroke: the jury is still out. *Nat. Med.* 16:959. 10.1038/nm0910-959 20823870

[B198] Vatine G. D. Barrile R. Workman M. J. Sances S. Barriga B. K. Rahnama M. (2019). Human iPSC-Derived Blood-Brain Barrier Chips Enable Disease Modeling and Personalized Medicine Applications. *Cell Stem Cell* 24 995.e–1005.e. 31173718 10.1016/j.stem.2019.05.011

[B199] Wang L. Sievert D. Clark A. E. Lee S. Federman H. Gastfriend B. D. (2021). A human three-dimensional neural-perivascular ‘assembloid’ promotes astrocytic development and enables modeling of SARS-CoV-2 neuropathology. *Nat. Med.* [Preprint]. 10.1038/s41591-021-01443-1 PMC8601037 34244682

[B200] Wang S. Cao C. Chen Z. Bankaitis V. Tzima E. Sheibani N. (2012). Pericytes regulate vascular basement membrane remodeling and govern neutrophil extravasation during inflammation. *PLoS One* 7:e45499–e45499. 10.1371/journal.pone.0045499 23029055 PMC3448630

[B201] Weis S. M. Cheresh D. A. (2013). A wake-up call for hibernating tumour cells. *Nat. Cell Biol.* 15 721–723. 10.1038/ncb2794 23817234

[B202] Wierenga C. E. Clark L. R. Dev S. I. Shin D. D. Jurick S. M. Rissman R. A. (2013). Interaction of age and APOE genotype on cerebral blood flow at rest. *J. Alzheimer’s Dis. JAD* 34 921–935. 10.3233/jad-121897 23302659 PMC4124882

[B203] Wimmer R. A. Leopoldi A. Aichinger M. Kerjaschki D. Penninger J. M. (2019a). Generation of blood vessel organoids from human pluripotent stem cells. *Nat. Protoc.* 14 3082–3100. 10.1038/s41596-019-0213-z 31554955

[B204] Wimmer R. A. Leopoldi A. Aichinger M. Wick N. Hantusch B. Novatchkova M. (2019b). Human blood vessel organoids as a model of diabetic vasculopathy. *Nature* 565 505–510. 10.1038/s41586-018-0858-8 30651639 PMC7116578

[B205] Winkler E. A. Bell R. D. Zlokovic B. V. (2011). Central nervous system pericytes in health and disease. *Nat. Neurosci.* 14 1398–1405. 10.1038/nn.2946 22030551 PMC4020628

[B206] Wörsdörfer P. I. T. Asahina I. Sumita Y. Ergün S. (2020). Do not keep it simple: recent advances in the generation of complex organoids. *J. Neural Transmiss.* 127 1569–1577. 10.1007/s00702-020-02198-8 32385575 PMC7577912

[B207] Xian X. Håkansson J. Ståhlberg A. Lindblom P. Betsholtz C. Gerhardt H. (2006). Pericytes limit tumor cell metastasis. *J. Clin. Invest.* 116 642–651. 10.1172/jci25705 16470244 PMC1361347

[B208] Xu J. Lazartigues E. (2020). Expression of ACE2 in Human Neurons Supports the Neuro-Invasive Potential of COVID-19 Virus. *Cell. Mol. Neurobiol.* 2020 1–5. 10.1007/s10571-020-00915-1 PMC7334623 32623546

[B209] Yemisci M. Gursoy-Ozdemir Y. Vural A. Can A. Topalkara K. Dalkara T. (2009). Pericyte contraction induced by oxidative-nitrative stress impairs capillary reflow despite successful opening of an occluded cerebral artery. *Nat. Med.* 15 1031–1037. 10.1038/nm.2022 19718040

[B210] Zhang S. Wan Z. Kamm R. D. (2021). Vascularized organoids on a chip: strategies for engineering organoids with functional vasculature. *Lab Chip* 21 473–488. 10.1039/d0lc01186j 33480945 PMC8283929

[B211] Zhao H. Chappell J. C. (2019). Microvascular bioengineering: a focus on pericytes. *J. Biol. Engine.* 13:26. 10.1186/s13036-019-0158-3 PMC6444752 30984287

[B212] Zheng X. Alsop D. C. Schlaug G. (2011). Effects of transcranial direct current stimulation (tDCS) on human regional cerebral blood flow. *Neuroimage* 58 26–33. 10.1016/j.neuroimage.2011.06.018 21703350 PMC3155947

[B213] Zhou W. Chen C. Shi Y. Wu Q. Gimple R. C. Fang X. (2017). Targeting Glioma Stem Cell-Derived Pericytes Disrupts the Blood-Tumor Barrier and Improves Chemotherapeutic Efficacy. *Cell Stem Cell* 21 591.e–603.e. 29100012 10.1016/j.stem.2017.10.002 PMC5687837

[B214] Zhu X. Raina A. K. Perry G. Smith M. A. (2004). Alzheimer’s disease: the two-hit hypothesis. *Lancet Neurol.* 3 219–226. 15039034 10.1016/S1474-4422(04)00707-0

[B215] Zimmermann K. W. (1923). Der feinere Bau der Blutcapillaren. *Zeitschrift für Anatomie Entwicklungsgeschichte* 68 29–109. 10.1007/bf02593544

[B216] Zlokovic B. V. (2011). Neurovascular pathways to neurodegeneration in Alzheimer’s disease and other disorders. *Nat. Rev. Neurosci.* 12 723–738. 22048062 10.1038/nrn3114 PMC4036520

[B217] Zou X. Chen K. Zou J. Han P. Hao J. Han Z. (2020). Single-cell RNA-seq data analysis on the receptor ACE2 expression reveals the potential risk of different human organs vulnerable to 2019-nCoV infection. *Front. Med.* 14:185–192. 10.1007/s11684-020-0754-0 32170560 PMC7088738

